# Impact of a 12‐week high‐intensity interval training intervention on cardiac structure and function after COVID‐19 at 12‐month follow‐up

**DOI:** 10.1113/EP092099

**Published:** 2024-09-11

**Authors:** Iben Elmerdahl Rasmussen, Mathilde Løk, Cody Garett Durrer, Anna Agnes Lytzen, Frederik Foged, Vera Graungaard Schelde, Josephine Bjørn Budde, Rasmus Syberg Rasmussen, Emma Fredskild Høvighoff, Villads Rasmussen, Mark Lyngbæk, Simon Jønck, Rikke Krogh‐Madsen, Birgitte Lindegaard, Peter Godsk Jørgensen, Lars Køber, Niels Vejlstrup, Bente Klarlund Pedersen, Mathias Ried‐Larsen, Morten Asp Vonsild Lund, Ronan M. G. Berg, Regitse Højgaard Christensen

**Affiliations:** ^1^ Centre for Physical Activity Research University Hospital Copenhagen – Rigshospitalet Copenhagen Denmark; ^2^ Department of Biomedical Sciences, Faculty of Health and Medical Sciences University of Copenhagen Copenhagen Denmark; ^3^ Department of Cardiology University Hospital Copenhagen – Rigshospitalet Copenhagen Denmark; ^4^ Department of Infectious Diseases University Hospital Copenhagen – Hvidovre Hospital Hvidovre Denmark; ^5^ Department of Pulmonary Medicine and Infectious Diseases North Zealand Hospital Hillerød Denmark; ^6^ Research Unit for Exercise Epidemiology, Department of Sports Science and Clinical Biomechanics University of Southern Denmark Odense Denmark; ^7^ Department of Clinical Physiology and Nuclear Medicine University Hospital Copenhagen – Rigshospitalet Copenhagen Denmark; ^8^ Neurovascular Research Laboratory, Faculty of Life Sciences and Education University of South Wales Pontypridd UK; ^9^ Department of Cardiology University Hospital Copenhagen – Herlev Hospital Herlev Denmark

**Keywords:** cardiac magnetic resonance imaging, diffusing capacity, exercise capacity, left ventricular mass, long‐COVID, rehabilitation

## Abstract

In patients previously hospitalised for COVID‐19, a 12‐week high‐intensity interval training (HIIT) intervention has previously been shown to increase left ventricular mass (LVM) immediately after the intervention. In the present study, we examined the effects of the same HIIT scheme on LVM, pulmonary diffusing capacity, symptom severity and functional capacity at 12‐month follow‐up. In this investigator‐blinded, randomised controlled trial, 12 weeks of a supervised HIIT scheme (4 × 4 min, three times a week) was compared to standard care (control) in patients recently discharged from hospital due to COVID‐19. At inclusion and at 12‐month follow‐up, LVM was assessed by cardiac magnetic resonance imaging (cMRI, primary outcome), while pulmonary diffusing capacity for carbon monoxide (*D*_LCOc_, secondary outcome) was examined by the single‐breath method. Symptom severity and functional status were examined by the Post‐COVID‐19 Functional Scale (PCFS) and King's Brief Interstitial Lung Disease (KBILD) questionnaire score. Of the 28 patients assessed at baseline, 22 completed cMRI at 12‐month follow‐up (12.4 ± 0.6 months after inclusion). LVM was maintained in the HIIT but not the standard care group, with a mean between‐group difference of 9.68 [95% CI: 1.72, 17.64] g (*P* = 0.0182). There was no differences in change from baseline to 12‐month follow‐up between groups in *D*_LCOc_ % predicted (−2.45 [−11.25, 6.34]%; *P* = 0.578). PCFS and KBILD improved similarly in the two groups. In individuals previously hospitalised for COVID‐19, a 12‐week supervised HIIT scheme resulted in a preserved LVM at 12‐month follow‐up but did not affect pulmonary diffusing capacity or symptom severity.

## Abstract (highlights)

- **What is the central question of this study?**
  In patients previously hospitalised for COVID‐19, a 12‐week high‐intensity interval training (HIIT) scheme increases left ventricular mass (LVM) immediately after the intervention: does this persist?
- **What is the main finding and its importance?**
  A 12‐week HIIT intervention led to preserved LVM at 12‐month follow‐up, while LVM decreased by ∼10 g in the standard care group. This indicates that HIIT may be useful as part of post‐COVID rehabilitation strategies for specifically targeting the detrimental effects of COVID‐19 on the heart.

## 1 INTRODUCTION

The post‐acute sequelae of the SARS‐CoV‐2 infection presents a multifaceted challenge. More than 25% of individuals experience persistent exercise intolerance at 12‐month follow‐up with a concomitant reduction in peak oxygen uptake (${\dot{V}}_{O_{2}\mathrm{peak}}$) after the acute phase of the infection. Furthermore, 15% of individuals continue to have symptoms at 12 months (Foged et al., 2021; Gesser et al., 2023; Vos et al., 2022). Even at 2‐year follow‐up a notable burden of symptoms persist, and the afflicted individuals do not always return to the same health status as the general population (Huang et al., 2022). Cardiopulmonary rehabilitation has been shown to be a safe and effective treatment for counteracting these detrimental effects of COVID‐19 (Kogel et al., 2023; Rooney et al., 2020). The harmful effects are thought to be multifactorial in origin, including the lung, heart and peripheral vasculature, and caused both by disease‐specific mechanisms and overt deconditioning (Ambrosino & Maniscalco, 2022; Baratto et al., 2021; Christensen & Berg, 2021; Evers et al., 2022; Katzenstein et al., 2022; Rinaldo et al., 2021; Sollini et al., 2021; Torres‐Castro et al., 2021).

We have recently reported that a supervised 12‐week high‐intensity interval training (HIIT) scheme leads to a ∼9 g increase in left ventricular mass (LVM) with a corresponding improvement of functional limitations immediately after the intervention, but with no effect on pulmonary diffusing capacity or other lung function metrics (Rasmussen et al., 2023). These findings are consistent with other training studies showing that rehabilitation programmes are effective in improving exercise capacity and alleviating COVID‐19‐associated symptoms and are superior to no intervention in patients surviving COVID‐19 (Ahmed et al., 2022; Barbara et al., 2022; Kogel et al., 2023). Despite these initial results, it is unknown whether these changes persist long‐term.

In the present study, which is a 12‐month follow‐up study of our previous randomised controlled trial on 12‐week supervised HIIT versus standard care (Rasmussen et al., 2023), we aim to investigate whether HIIT has long‐term effects on cardiopulmonary function in individuals previously hospitalised for COVID‐19. We hypothesised that HIIT allocation would be associated with (1) a higher LVM, (2) a similar pulmonary diffusing capacity, and (3) fewer symptoms and better functional capacity at 12‐month follow‐up than in those allocated to standard care.

## 2 METHODS

### 2.1 Ethics

The study was approved by The Research Ethics Committee of the Capital Region of Denmark (H‐20033733 with amendment 75068). The study conformed to the *Declaration of Helsinki*, and all participants provided oral and written informed consent prior to participation.

### 2.2 Study design and participants

The present study was a randomised controlled trial on previously hospitalised COVID‐19 patients; the study protocol has been published elsewhere (Rasmussen et al., 2021), as have baseline versus 3‐month follow‐up findings (Rasmussen et al., 2023). The enrolment period occurred from 1 February 2021 to 1 February 2022. The following inclusion criteria were applied: (1) hospitalisation with SARS‐CoV‐2 confirmed by a polymerase chain reaction within the previous 6 months; (2) age ≥40 years; and (3) a maximum requirement of 10 L/O_2_/min during hospitalisation. The exclusion criteria were: (1) diagnosis of acute myocarditis during COVID‐19 hospitalisation, (2) present atrial fibrillation/flutter, (3) ongoing treatment with an interleukin‐6 receptor inhibitor up to 1 month prior to inclusion, (4) critical physical injury or dysfunction, including angina pectoris and heart failure, (5) absolute contraindications for cardiac magnetic resonance imaging (cMRI). Participants were recruited at hospital discharge or contacted by telephone immediately after. All experimental procedures and interventions were carried out at the Centre for Physical Activity Research (CFAS), and The Department of Cardiology (both at Rigshospitalet, Denmark).

### 2.3 Randomisation, blinding and allocation concealment

Participants were invited to baseline visits prior to a 1:1 randomisation to either the intervention group (HIIT) or the control group (standard care), stratified by sex. The randomisation procedure was conducted by two researchers not involved in the experimental procedures. First by using a computer‐generated block schedule (research randomizer, version 4), a randomised sequence was stored on a password‐protected computer. Following completion of baseline examination, the independent researcher administered the allocation procedure and revealed the allocation assignment to a clinician (I.R.), who contacted the participants by telephone. The assessors of imaging and lung function outcomes were blinded to participant allocation. Due to the nature of the study, participants could not be blinded to intervention allocation.

### 2.4 Intervention

The intervention has been described in detail elsewhere (Rasmussen et al., 2021). Briefly, it consisted of a 12‐week individually supervised 4 × 4 HIIT scheme with three weekly sessions of 38 min including a 10‐min warm‐up on a bicycle ergometer by an experienced kinesiologist (Foged et al., 2021). During each 4 min interval, the participant was encouraged to keep the intensity on at least 85% of maximal heart rate (HR_max_), as determined by a cardiopulmonary exercise test (CPET) at baseline. These intervals were interspersed by 3 min active recovery breaks at 50–70% of HR_max_. The whole session was preceded by a 10 min warm‐up and ended with a cool‐down. Protocol adherence was fulfilled when the participant spent ≥25% of the training time at ≥85% of HR_max_ (zone 5), as determined by a telemetry system (Polar RS400; Polar, Kempele, Finland) (Rasmussen et al., 2021).

### 2.5 Outcomes

Outcomes were assessed as the between‐group change from baseline to 12‐month follow‐up. The primary outcome was change in LVM in grams. The key secondary outcome was between‐group change in pulmonary diffusing capacity for carbon monoxide corrected for haemoglobin (*D*_LCOc_). The secondary outcomes were between‐group change in ${\dot{V}}_{O_{2}\mathrm{peak}}$, as well as the post‐COVID‐19 function scale (PCFS) and King's Brief Interstitial Lung Disease (KBLID) questionnaire scores. Furthermore, the following exploratory outcomes were examined: LVM indexed for body surface area (BSA), left ventricular stroke volume (LVSV), left ventricular end‐diastolic volume (LVEDV), left ventricular ejection fraction (LVEF), residual lung volume (RV), total lung capacity (TLC), forced expiratory volume in 1 s (FEV_1_), forced vital capacity (FVC), fat mass and fat‐free mass, as well as leg‐ and chest‐press one‐repetition maximum (1RM).

### 2.6 Measurements

#### 2.6.1 Cardiac MRI

All cMRI scans were conducted in an observer‐blinded fashion at the Department of Cardiology, Rigshospitalet, Denmark, using a dedicated 1.5 T cardiac scanner (MAGNETOM Aera; Siemens Healthcare, Erlangen, Germany) as previously described elsewhere (Rasmussen et al., 2021, 2023). In brief, imaging was performed during expiratory breath‐hold and was ECG‐gated to assess LVM, volumes and tissue characterization, using steady‐state free precession images and T1/T2 mapping across ventricular slices. Evaluations followed current guidelines (Schulz‐Menger et al., 2020). BSA for indexing of LVM was estimated by the Mosteller formula (Mosteller, 1987).

#### 2.6.2 Lung function

Lung function testing included dynamic spirometry (for FEV_1_ and FVC), body plethysmography (for TLC and RV), and single‐breath carbon monoxide uptake (for *D*_LCOc_) in accordance with current clinical guidelines (Bhakta et al., 2023; Graham et al., 2019). Values were reported as percentage of predicted (%pred) according to height, sex and age as appropriate (Stanojevic et al., 2022).

#### 2.6.3 Symptoms and health‐related quality of life

The KBILD questionnaire included three domains (Prior et al., 2019): psychological (KBILD‐P), breathlessness and activities (KBILD‐B) and chest symptoms (KBILD‐C), which were all compiled in a total score (KBILD‐T), with 100 being the best and 0 the worst score. PCFS questionnaire scores ranged from grade 0 (best) to grade 4 (worst) (Klok et al., 2020).

#### 2.6.4 Peak oxygen uptake

A graded CPET was performed on an ergometer bicycle (Monark LC4, Monark Exercise AB, Vansbro, Sweden). Following a 5 min warm‐up at 50 W (male) or 30 W (female), the participant was verbally encouraged to maintain a cadence of 70–100 revolutions per min (rpm), while the workload was progressively increased by 20 W/min until volitional exhaustion or failure to maintain more than 60 rpm. ${\dot{V}}_{O_{2}}$ was measured by indirect calorimetry (Quark CPET, Cosmed, Rome, Italy), and heart rate was monitored simultaneously. The CPET was considered valid for determining maximal ${\dot{V}}_{O_{2}}$ when (1) a plateau >30 s in ${\dot{V}}_{O_{2}}$ could be visually identified, (2) the respiratory exchange ratio was >1.1, and (3) the BORG scale was rated as >17. If only two of these could be attained, the highest 20‐s mean ${\dot{V}}_{O_{2}}$ during the CPET was defined as ${\dot{V}}_{O_{2}\mathrm{peak}}$. Absolute ${\dot{V}}_{O_{2}\mathrm{peak}}$ (mL/min) and relative ${\dot{V}}_{O_{2}\mathrm{peak}}$ (mL/min/kg body weight) were reported.

#### 2.6.5 Strength testing

Upper and lower body strength were assessed by 1RM tests, defined as the maximum amount of weight that can be lifted once with proper form through the full range of motion, using chest‐press and leg‐press machines (Technogym Runrace, Gambettola, Italy) (Rasmussen et al., 2021).

#### 2.6.6 Dual‐energy X‐ray absorptiometry

A total body dual energy X‐ray absorptiometry (Lunar Prodigy, GE Healthcare, Madison, WI, USA; encore software version 14, 10, 022) was used to estimate total fat mass, fat mass percentage and fat free mass.

### 2.7 Safety and adverse events

Adverse events (AE) were only documented during the intervention period and are reported elsewhere (Rasmussen et al., 2023). All cMRI scans were assessed by an independent experienced cardiologist to screen for myocarditis and other ancillary findings.

### 2.8 Sample size

The sample size was based on an anticipated within‐group increase of LVM 7% (SD 10%) in the HIIT group and 0% (SD 10%) in the control group (Rasmussen et al., 2021; 2023). Inclusion of 24 participants in each group would provide a statistical power ((1 − β) × 100%) of 86% with a two‐sided significance level of 0.05 using an analysis of covariance assuming a correlation between the baseline and follow‐up measurements of 0.6 (Rasmussen et al., 2021).

### 2.9 Statistical analysis

The statistical analysis plan is provided elsewhere and was published online in detailed form before any analyses were conducted (https://doi.org/10.17605/OSF.IO/ANW3D). All analyses were performed using R (Version 4.3.0) (R Core Team, 2021).

Continuous outcomes were analysed using constrained baseline longitudinal analyses via linear mixed models and presented as mean differences with 95% CIs (Coffman et al., 2016), which allows all participants with at least one measurement (baseline or follow‐up) to be included in the analysis. Missing data were assumed to be missing at random. Linear models were specified using the lme4 package (Version 1.1‐35.1 https://github.com/lme4/lme4/) and included fixed effects for time point (baseline, 3 months or 12 months), treatment (coded as ‘0’ if time point = baseline or allocation = control and coded ‘1’ otherwise), sex (male or female), a timepoint × treatment interaction, as well as a random intercept for participants. Estimated marginal means and mean differences were calculated via the emmeans package (Version 1.8.9; https://github.com/rvlenth/emmeans). Model specification was assessed visually using normal probability plots and residuals versus fitted values plots. When warranted, nonparametric bootstrap analyses with 2000 resamples with replacement (Davison & Hinkley, 1997) were performed (boot package, version 1.3‐28.1), and bias‐corrected and accelerated 95% confidence intervals were then calculated (https://cran.r‐project.org/package=boot).

The intention‐to‐treat (ITT) population for a given outcome included any participant with an assessment at baseline and/or follow‐up. Per protocol (PP) analysis was performed by excluding participants who did not fulfil the predefined adherence criteria. Additionally, a sensitivity analysis was performed excluding those in the control group who started regular exercise outside of this study. Only complete cases for each outcome were included in the PP analysis and sensitivity analysis.

Results are reported in accordance with the Consolidated Standards of Reporting Trials (CONSORT) (Moher et al., 2012), and presented as mean ± SD or median [IQR, 25th and 75th percentile] (baseline) and estimated marginal means or mean difference [95% CI] (follow‐up) as appropriate, apart from PCFS, which is presented descriptively. Statistical significance was set at a two‐sided *P* ≤ 0.05.

## 3 RESULTS

### 3.1 Participants

**FIGURE 1:**
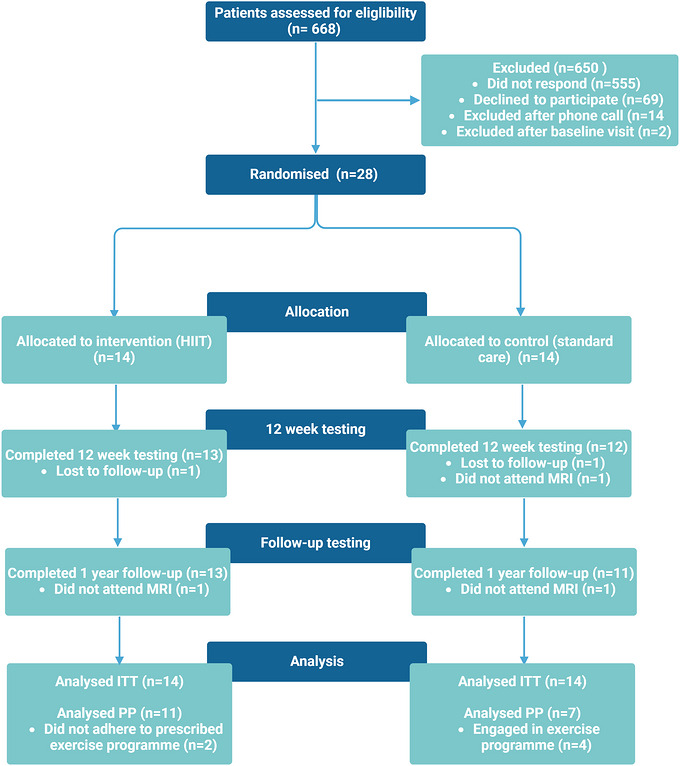
Study flow chart for participants. Four participants did not attend at 12 months as it was not possible to get in contact. One participant did not attend MRI at 12 months for no particular reason.

A CONSORT flow diagram is provided in Figure 1. Of the 28 patients invited from the main study, 24 completed the 12‐month follow‐up, which was completed 12.4 ± 0.6 months after baseline measurements. A total of 28 participants were included in the final ITT analysis at 12 months (HIIT: *n* = 14, control: *n* = 14). Thus, a total of 22 participants completed cMRI at 12 months. Table 1 summarises the descriptive characteristics of the study participants at baseline.

**TABLE 1:** Baseline characteristics at inclusion (0 month).

	Control (*n* = 14)	Intervention (*n* = 14)	Total (*n* = 28)
Demographics			
Sex, men/total (*n*)	9/14	10/14	19/28
Age (years)	56.6 ± 9	58 ± 11.2	57.3 ± 10
BMI (kg/m^2^)	32.8 ± 10	29.2 ± 4.9	31 ± 7.9
Fat mass (%)	37.4 ± 9.1	36.5 ± 8.1	37 ± 8.5
Lean body mass (kg)	58.3 ± 15.7	54.0 ± 13.4	56.1 ± 14.7
Duration of hospitalisation (days)	6 [5, 9]	8 [6, 10]	7 [6, 10]
Bedrest in total (days)	22 [16, 27]	17 [13, 21]	20 [15, 24]
Time since discharge (days)	42 [32, 63]	42 [33, 95]	42 [32, 70]
Systolic BP (mmHg)	136 ± 11	135 ± 16	135 ± 14
Diastolic BP (mmHg)	84 ± 7	83 ± 11	83 ± 9
Resting HR (BPM)	71 ± 12	71 ± 12	71 ± 12
Resting haemoglobin (mmol/L)	8.4 ± 0.9	8.4 ± 0.9	8.4 ± 0.9
Physical activity level before hospitalisation (*n*)			
Very light or light	9/14	2/14	11/28
Moderate	4/14	7/14	11/28
Active or very active	1/14	5/14	6/28
Physical activity level after hospitalisation (*n*)			
Very light or light	12/14	7/14	19/28
Moderate	2/14	5/14	7/28
Active or very active	0/14	1/14	1/28
Lung function			
FEV_1_%pred	98.2 ± 11.3	101.1 ± 17.1	99.7 ± 14.7
FVC%pred	97.1 ± 12.2	100.2 ± 19	98.7 ± 16.1
TLC%pred	87.4 ± 10.7	88.1 ± 17.3	87.8 ± 14.5
RV%pred	82.0 ± 19.3	92.8 ± 24.0	87.36 ± 22.5
*D*_LCOc_%pred	80.7 ± 21.7	81.7 ± 16.1	81.2 ± 19.0
Exercise capacity			
${\dot{V}}_{O_{2}\mathrm{peak}}$ (mL/min)	2210 ± 962	2052 ± 836	2131 ± 903
${\dot{V}}_{O_{2}\mathrm{peak}}$ (mL/min/kg)	22.3 ± 7.8	22.3 ± 7.8	22.8 ± 7.6

*Note*: Data are presented as mean ± SD and median [25th percentile, 75th percentile]. Abbreviations: BP, blood pressure; *D*_LCOc_, carbon monoxide diffusing capacity corrected for haemoglobin; FEV_1_, forced expiratory volume in 1 s; FVC, forced vital capacity; RV, residual volume; TLC, total lung capacity; ${\dot{V}}_{O_{2}}$ peak, peak oxygen uptake.

Protocol adherence has been reported elsewhere (Rasmussen et al., 2023). All results from the ITT, PP and sensitivity analysis are available in Supporting information Supplements S1, S2 and S3, respectively. Results of the ITT are presented below.

### 3.2 Cardiac structure and function

**FIGURE 2:**
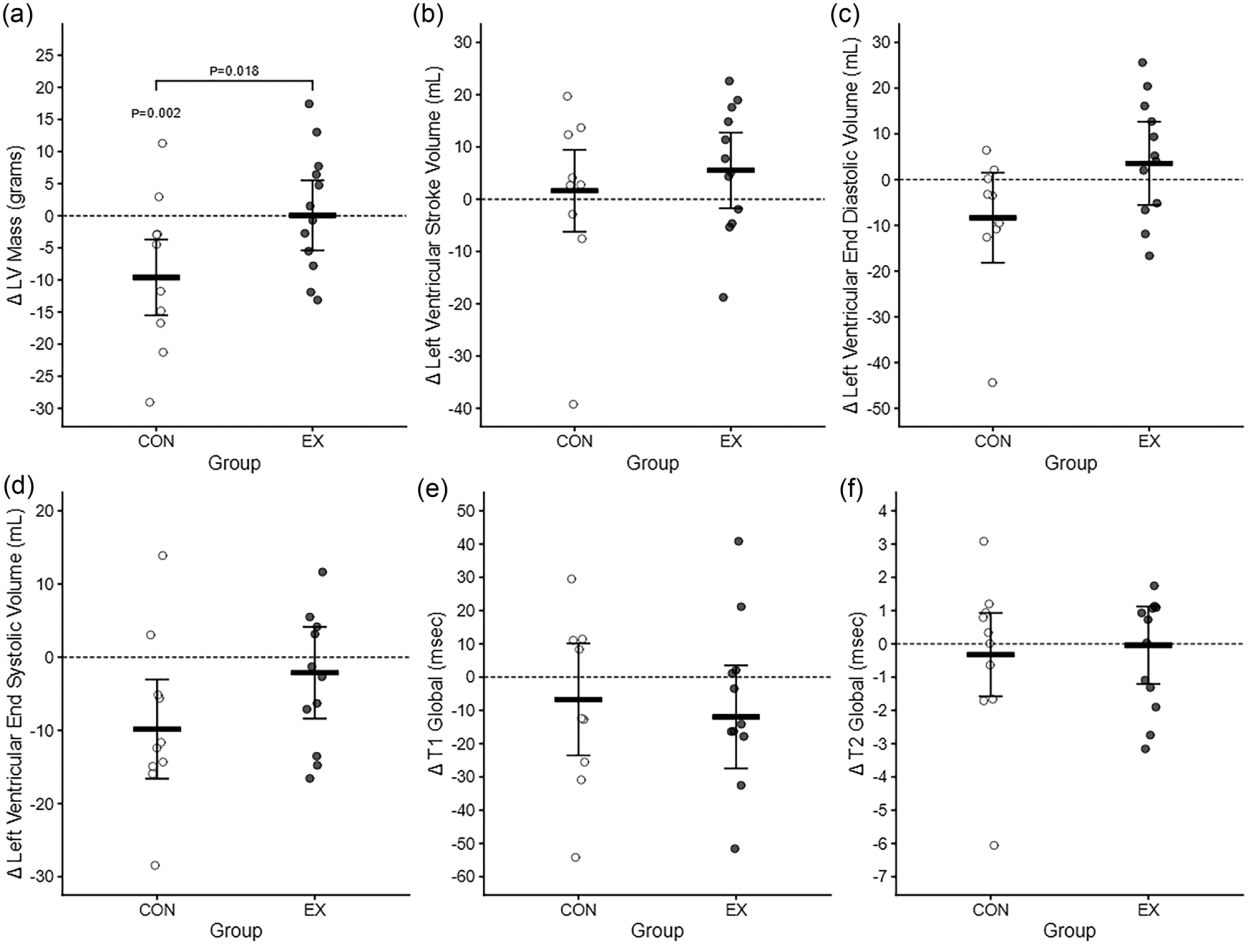
Effect of high‐intensity interval training (HIIT) on left ventricular mass (LVM) and volumes, and T1/T2 mapping. Changes LVM (a), left ventricular stroke volume (LVSV, b), left ventricular end‐diastolic volume (LVEDV, c), left ventricular end‐systolic volume (LVESD) as well as T1 (d) and T2 (e) mapping from baseline (pre) to 12 months (post). Data are provided for the intention‐to‐treat analysis. White is the control (standard care) group (*n* = 13) and black is the intervention (HIIT) group (*n* = 13). Data are presented as within‐group mean difference [95% CI] from baseline. *P* < 0.05 versus baseline and *P* < 0.05 versus between‐group.

At 12‐month follow‐up, LVM was preserved in the HIIT group compared to the controls with a between‐group difference in change of 9.68 [1.72, 17.64] g (*P *= 0.0182) (Figure 2a). LVEDV and LVESV showed a similar pattern (LVEDV: 11.85 [−1.31, 25.00] mL, *P *= 0.0765 and LVESV: 7.7 [−1.21, 16.62] mL, *P *= 0.0891), while LVSV did not change in either group (between‐group difference −3.91 [−6.50, 14.32] mL, *P *= 0.455) (Figure 2b,c). There was no change within‐ or between‐groups in global T1 or T2 values (Figure 2d,e). Other relevant cMRI‐derived parameters, including BSA‐indexing are provided in Table 2.

**TABLE 2:** Effect of high‐intensity interval training (HIIT) on selected cardiac magnetic resonance imaging‐based parameters.

	Baseline (0 month)	12 months		12‐month treatment effect	
		Control	HIIT		
	Estimated mean [95% CI]	Estimated mean [95% CI]	Estimated mean [95% CI]	Between‐group mean [95% CI]	*P*
LVM/BSA (g/m^2^)	55.70 [52.51 to 58.89]	52.10 [48.15 to 56.05]	54.61 [50.81 to 58.42]	2.51 [−1.71 to 6.73]	0.24
LVEDV/BSA (mL/m^2^)	65.44 [61.12 to 69.75]	61.63 [56.24 to 67.01]	65.93 [60.75 to 71.11]	4.30 [−1.52 to 10.12]	0.144
LVSV/BSA (mL/m^2^)	41.21 [38.04 to 44.38]	42.19 [38.13 to 46.25]	43.12 [39.24 to 47.01]	0.94 [−3.62 to 5.50]	0.682
LVESV/BSA (mL/m^2^)	24.25 [21.71 to 26.78]	19.50 [16.06 to 22.94]	22.87 [19.61 to 26.14]	3.37 [−0.77 to 7.51]	0.108
LVEF (%)	63.00 [60.30 to 65.71]	68.50 [64.68 to 72.32]	65.00 [61.39 to 68.60]	−3.50 [−8.27 to 1.26]	0.146
CI (L/min/m^2^)	2.95 [2.75 to 3.16]	3.10 [2.79 to 3.41]	2.90 [2.61 to 3.19]	−0.19 [−0.60 to 0.21];	0.343
PER (mL/s)	506.55 [462.62 to 550.47]	547.73 [482.89 to 612.56]	446.79 [386.01 to 507.58]	−100.93 [−184.48 to −17.39]	0.0187
PFR (mL/s)	421.97 [371.86 to 472.08]	412.41 [341.92 to 482.91]	415.29 [348.72 to 481.86]	2.87 [−84.74 to 90.49]	0.948

*Note*: Data are expressed as estimated marginal mean [95% CI] for each group, difference within‐groups, and difference between‐group [95% CI]. Baseline means (0 month) are constrained to be the same. Data are provided for the intention‐to‐treat analysis (control group [standard care], *n* = 14; intervention group [HIIT], *n* = 14). Abbreviations: CI, cardiac output/body surface area; EF, ejection fraction; LVM/BSA, left ventricular mass/body surface area; LVEDV, left ventricular end‐diastolic volume, LVSV, left ventricular stroke volume; LVESV/BSA, left ventricular end‐systolic volume/body surface area; LVEDV/BSA, left ventricular end‐diastolic volume/body surface area; LVSV/BSA, left ventricular stroke volume/body surface area; HR, heart rate; PER, peak ejection rate; PFR, peak filling rate.

### 3.3 Lung function

*D*_LCOc_%pred, FEV_1_%pred, FVC%pred and TLC%pred increased similarly in both groups, while RV%pred only increased in the control group (Table 3). At 12 months, *D*_LCOc_%pred was reduced in 4/23 (17%; HIIT: *n* = 2, standard care: *n* = 2) and RV%pred in 1/23 (4%; HIIT: *n* = 1, standard care: *n* = 0), while there were no participants with reduced TLC%pred, FEV_1_%pred or FVC%pred at follow‐up.

**TABLE 3:** Effect of high‐intensity interval training (HIIT) on lung function, CPET and strength, body composition and KBILD‐scores.

	Baseline	12 months		12‐month treatment effect	
		Control	HIIT		
	Estimated mean [95% CI]	Estimated mean [95% CI]	Estimated mean [95% CI]	Between‐group mean [95% CI]	*P*
Lung function					
FEV_1_%pred	99.19 [93.20 to 105.18]	110.93 [103.31 to 118.56]	111.31 [103.89 to 118.72]	0.37 [−8.31 to 9.05]	0.932
FVC%pred	98.55 [92.59 to 104.51]	111.54 [104.07 to 119.02]	109.16 [101.88 to 116.45]	−2.38 [−10.71 to 5.95]	0.570
TLC%pred	87.42 [81.82 to 93.01]	99.37 [92.34 to 106.40]	97.37 [90.53 to 104.22]	−2.00 [−9.86 to 5.86]	0.613
RV%pred	87.39 [79.70 to 95.08]	97.65 [87.03 to 108.28]	93.53 [83.30 to 103.76]	−4.12 [−17.41 to 9.16]	0.537
*D*_LCOc_%pred	78.78 [72.67 to 84.89]	88.09 [80.37 to 95.80]	85.63 [78.11 to 93.15]	−2.45 [−11.25 to 6.34]	0.578
CPET and strength					
Absolute ${\dot{V}}_{O_{2}\mathrm{peak}}$ (mL/min)	1909.33 [1643.02 to 2175.64]	2087.55 [1759.28 to 2415.82]	2284.82 [1984.96 to 2584.67]	197.27 [−108.30 to 502.84]	0.200
Relative ${\dot{V}}_{O_{2}\mathrm{peak}}$ (mL/kg/min)	21.63 [19.10 to 24.16]	23.65 [20.37 to 26.93]	24.77 [21.84 to 27.71]	1.12 [−2.17 to 4.42]	0.497
* W*_max_ (peak power output)	153.90 [127.39 to 180.42]	204.62 [167.09 to 242.16]	194.78 [161.39 to 228.18]	−9.84 [−53.32 to 33.64]	0.652
Chest press (1RM, kg)	41.82 [34.66 to 48.98]	44.24 [36.37 to 52.11]	45.44 [37.59 to 53.29]	1.20 [−5.05 to 7.45]	0.701
Leg press (1RM, kg)	135.48 [116.80 to 154.15]	153.92 [130.28 to 177.55]	145.45 [122.54 to 168.35]	−8.47 [−34.78 to 17.84]	0.521
Body composition					
Total fat mass (g)	33560.42 [27649.75, 39471.08]	33344.33 [27374.05, 39314.61]	34309.94 [28347.16, 40272.71]	965.60 [−688.69, 2619.89]	0.246
Total fat mass (%)	38.71 [35.83, 41.58]	38.46 [35.51, 41.40]	38.17 [35.23, 41.10]	−0.29 [−1.54, 0.96]	0.64
Android fat mass (g)	3403.96 [2783.07, 4024.86]	3414.12 [2778.32, 4049.91]	3546.28 [2912.37, 4180.20]	132.17 [−135.16, 399.49]	0.325
Android fat mass (%)	47.15 [44.01 to 50.29]	47.21 [43.94 to 50.47]	47.23 [43.98 to 50.47]	0.02 [−1.70 to 1.74]	0. 980
Gynoid fat mass (g)	4484.00 [3847.24 to 5120.77]	4459.88 [3799.51 to 5120.25]	4678.43 [4021.04 to 5335.81]	218.55 [−121.55 to 558.65]	0. 202
Gynoid fat mass (%)	41.75 [38.89 to 44.61]	41.17 [38.19 to 44.16]	40.72 [37.75 to 43.68]	−0.46 [−2.06 to 1.15]	0.569
Fat free mass (g)	51842.61 [47978.63 to 55706.58]	51922.99 [47972.42 to 55873.57]	54420.54 [50480.89 to 58360.19]	2497.54 [889.29 to 4105.80]	0.00305
Weight (kg)	89.71 [79.83 to 99.59]	90.23 [80.26 to 100.20]	93.84 [83.89 to 103.79]	3.61 [1.14 to 6.07]	0.00503
BMI (kg/m^2^)	30.40 [27.13 to 33.66]	30.51 [27.22 to 33.80]	31.66 [28.37 to 34.95]	1.15 [0.35 to 1.95]	0.00589
Bone mass density (g/cm^3^)	1.23 [1.19 to 1.26]	1.22 [1.18 to 1.26]	1.23 [1.19 to 1.27]	0.01 [−0.01 to 0.03]	0.430
KBILD scores					
KBILD‐P	74.41 [68.91 to 80.69]	88.28 [79.05 to 95.67]	89.88 [82.15 to 99.25]	1.60 [−9.51 to 15.72]	N/A
KBILD‐B	47.03 [40.11 to 54.49]	67.18 [57.77 to 77.49]	66.34 [57.75 to 79.43]	−0.84 [−11.69 to 14.79]	N/A
KBILD‐C	78.88 [72.36 to 84.25]	91.93 [87.10 to 99.72]	91.21 [79.29 to 99.33]	−0.72 [−18.43 to 9.25]	N/A
KBILD‐T	64.37 [59.10 to 69.65]	78.68 [71.78 to 85.58]	77.45 [70.77 to 84.14]	−1.23 [−9.37 to 6.92]	0.765

*Note*: Data are expressed as estimated marginal mean [95% CI] for each group, mean difference within‐groups, and mean difference between‐group [95% CI]. Baseline means are constrained to be the same. Data are provided for the intention‐to‐treat analysis (control group [standard care], *n* = 14; intervention group [HIIT], *n* = 14). Abbreviations: CPET, cardiopulmonary exercise testing; *D*_LCOc_, pulmonary diffusing capacity for carbon monoxide corrected for haemoglobin; FEV1, forced expiratory volume 1 s; FVC, forced vital capacity; KBILD‐B, King's Brief Interstitial Lung Disease, breathlessness and activity domain; KBILD‐C, King's Brief Interstitial Lung Disease, chest symptoms domain; KBILD‐P, King's Brief Interstitial Lung Disease, psychological domain; KBILD‐T, King's Brief Interstitial Lung Disease, total score; RV, residual volume; TLC, total lung capacity; ${\dot{V}}_{O_{2}\mathrm{peak}}$, peak oxygen consumption, 1RM, 1 repetition max.

### 3.4 Functional capacity and health‐related quality of life

Both groups increased in total KBILD scores, without any between‐group differences (Table 3). In terms of PCFS, the majority had no functional limitations at 12 months (HIIT: 76.9%, control: 63.6%; Table 4).

**TABLE 4:** Effect of high‐intensity interval training (HIIT) on the Post COVID‐19 Functional Scale.

	Control		HIIT	
Time‐point	Baseline	12 months	Baseline	12 months
0	5 (35.7%)	7 (63.6%)	4 (28.6%)	10 (76.9%)
1	2 (14.3%)	1 (9.1%)	2 (14.3%)	1 (7.1%)
2	5 (35.7%)	3 (27.3%)	4 (28.6%)	1 (7.1%)
3	2 (14.3%)	0 (0.0%)	2 (14.3%)	1 (7.1%)
4	0 (0.0%)	0 (0.0%)	2 (14.3%)	0 (0.0%)

*Note*: Data are presented descriptively with *n* (%) for the control group (standard care, *n* = 11) and intervention group (HIIT, *n* = 13). Grade 0 reflects no functional limitations, whereas from grade 1 upwards, symptoms, pain or anxiety are present to an increasing degree.

### 3.5 Other secondary and exploratory outcomes

Relative ${\dot{V}}_{O_{2}\mathrm{peak}}$ increased in the HIIT group, by 3.14 [1.04, 5.25] mL/kg/min (*P *= 0.00436) and by 2.02 [−0.59, 4.63] mL/kg/min (*P *= 0.126) in the control group with a between‐group difference of 1.12 [−2.17, 4.42] mL/kg/min (*P *= 0.497) at 12 months; a similar pattern was shown for absolute ${\dot{V}}_{O_{2}\mathrm{peak}}$. Fat free mass and body weight increased significantly in the HIIT group compared to the control group by 2497.5 [889.3, 4105.8] g (*P *= 0.00305) and 3.61 [1.14, 6.07] kg (*P *= 0.00503), respectively, but there were no other notable between‐group changes in body composition, peak power output (*W*_max_) or strength.

### 3.6 Safety

Two individuals in the HIIT group showed signs of myocarditis on cMRI, both at baseline and at 12‐month follow‐up, but without symptoms of other clinical abnormalities. One individual in the control group showed mild cardiac hypertrophy at 12‐month follow‐up. All were referred for adequate clinical assessment, and in none of the cases did this lead to any clinical intervention. No other ancillary findings were identified on cMRI at 12 months.

## 4 DISCUSSION

The present randomised controlled study examined the long‐term effects of a 12‐week HIIT scheme on cardiopulmonary function in individuals previously hospitalised with COVID‐19, and showed that this was associated with a preserved LVM at 12 months when compared to controls in whom LVM decreased. Lung function metrics, notably including *D*_LCOc_, as well as functional capacity and health‐related quality of life (HRQoL) improved in both groups with no differences between groups.

LVM was the primary outcome of the study and exhibited a between‐group difference of change of approximately 10 g at 12 months from baseline in the control group, indicating that HIIT may preserve LVM after COVID‐19 while it gradually decreased in controls. This is further supported by the between‐group differences observed in LVEDV and LVESV. Furthermore, in both the PP and the sensitivity analysis, LVSV increased, such that it became higher in the HIIT group (PP: 8.49 [0.63, 16.36] mL, *P *= 0.035 and sensitivity analysis: 8.82 [2.37, 15.27] mL, *P *= 0.009). While these analyses must be interpreted with caution, this increase in LVSV, albeit measured at rest, likely comprises the mechanistic link to the ${\dot{V}}_{O_{2}\mathrm{peak}}$ increase, which is consistent with the physiological adaptations to HIIT in healthy individuals (Karlsen et al., 2017). This is also supported by prior studies using exercise echocardiography and magnetic resonance‐enhanced CPET to show an attenuated LVSV response to exercise as a critical limiting factor for ${\dot{V}}_{O_{2}\mathrm{peak}}$ in individuals hospitalised with COVID‐19 (Brown et al., 2022; Szekely et al., 2021). Nevertheless, it must be noted that ${\dot{V}}_{O_{2}\mathrm{peak}}$ remained below the lower limit of normal in a substantial proportion of our participants, regardless of treatment allocation, which is also consistent with previous findings (Szekely et al., 2021). This prolonged reduction is attributable not solely to limitations in LVSV but likely also a combination of diminished pulmonary diffusing capacity (Katzenstein et al., 2022; Torres‐Castro et al., 2021), vascular inflammation and dysfunction (Ambrosino & Maniscalco, 2022; Christensen & Berg, 2021; Sollini et al., 2021), as well as compromised peripheral oxygen uptake kinetics (Baratto et al., 2021; Evers et al., 2022).

Of note, the HIIT was associated with a preserved LVM from baseline at 12‐month follow‐up, while controls showed a decrease. The cause of this remains speculative, but it may either reflect disease‐specific effects on the heart that may be counteracted by HIIT (Rahmati et al., 2023), or alternatively that the control group merely remained physically inactive with a consequent gradual reduction in LVM. Indeed, the HIIT group did remain more physically active than the control group right after the intervention period (Rasmussen et al., 2023), but unfortunately, we did not obtain data on physical activity levels in the meantime. Nevertheless, exertional dyspnoea and other post‐COVID‐19 symptoms have previously been reported to lead to changes in physical activity patterns, where avoidance behaviour results in deconditioning (Shelley et al., 2021), a process thought to be an important component of the long COVID syndrome (Ambrosino & Maniscalco, 2022; Rinaldo et al., 2021). This may be prevented by targeted interventions, as has been previously shown for inspiratory muscle training, leading to an increase in physical activity and ${\dot{V}}_{O_{2}\mathrm{peak}}$ (McNarry et al., 2022). Furthermore, it has previously been reported that COVID‐19 patients who participated in a 4‐week strength and endurance training programme were more physical active 6 months after an exercise intervention compared to a control group (Kogel et al., 2023). The same might be true here, which could also explain the reported greater fat free mass and concomitant body weight in the HIIT group at 12 months, which seems to follow the change in LVM in the two groups closely, as indicated by the lack of statistically significant differences when using BSA‐indexed measures. However, it must be noted that no distinct patterns in functional capacity or HRQoL that would unequivocally support this were observed, as PCFS and KBILD scores improved similarly in the two groups.

There are some limitations of this study. We lack data on changes in physical activity levels between 12 weeks and 12 months. Furthermore, the patients were included from the first waves of the pandemic, so the findings may not be directly applicable to patients infected by later SARS‐CoV‐2 variants. The study furthermore faced a selection bias, as only patients willing to participate can be randomised, thus potentially favouring individuals with an a priori better prognosis.

In conclusion, a gradual decrease in LVM appears over the first 12 months after hospitalisation for COVID‐19, but this may be prevented by a 12‐week supervised HIIT scheme. Meanwhile, pulmonary diffusing capacity and other clinical lung function metrics, as well as functional capacity and HRQoL seem to improve spontaneously after COVID‐19 without any additional effect of HIIT.

## AUTHOR CONTRIBUTIONS

Iben Elmerdahl Rasmussen: Data collection; data analysis; data interpretation; first draft; revisions. Mathilde Løk: Data collection; data analysis; data interpretation; revisions. Cody Garett Durrer: Data analysis; data interpretation; revisions. Anna Agnes Lytzen: Data analysis; revisions. Frederik Foged: Data collection; data interpretation; revisions. Vera Graungaard Schelde: Data collection; data interpretation; revisions. Josephine Bjørn Budde: Data collection; data interpretation; revisions. Rasmus Syberg Rasmussen: Data collection; data interpretation; revisions. Emma Fredskild Høvighoff: Data collection; data interpretation; revisions. Villads Rasmussen: Design; data collection; data interpretation; revisions. Mark Lyngbæk: Data collection; data interpretation; revisions. Simon Jønck: Data collection; data interpretation; revisions. Rikke Krogh‐Madsen: Data collection; data interpretation; revisions. Birgitte Lindegaard: Data collection; data interpretation; revisions. Peter Godsk Jørgensen: Data interpretation; revisions. Lars Køber: Data interpretation; revisions. Niels Vejlstrup: Data interpretation; revisions. Bente Klarlund Pedersen: Data interpretation; revisions. Mathias Ried‐Larsen: Design; data analysis; data interpretation; revisions. Morten Asp Vonsild Lund: Data collection; data analysis; data interpretation; revisions. Ronan M. G. Berg: Conception; design; data analysis; data interpretation; first draft; revisions; supervision. Regitse Højgaard Christensen: Conception; design; data analysis; data interpretation; revisions; supervision. All authors approved the final version of the manuscript and agree to be accountable for all aspects of the work in ensuring that questions related to the accuracy or integrity of any part of the work are appropriately investigated and resolved.

## CONFLICT OF INTEREST

None declared.

## Supporting information

Supplement 1. Full ITT analysis.

Supplement 2. PP analysis.

Supplement 3. Sensitivity analysis.

## Data Availability

The raw data supporting the results in this study have been uploaded to the Open Science Framework (OSF) and can be accessed at DOI https://doi.org/10.17605/OSF.IO/D6429 upon reasonable request to the corresponding author.
