# Supplementary material for: Impact of a 12‐week high‐intensity interval training intervention on cardiac structure and function after COVID‐19 at 12‐month follow‐up
Source: Exp Physiol. 2024 Sep 11;111(6):2975–86. doi: 10.1113/EP092099 (PMC13238539; doi:10.1113/EP092099)
Supplement: Supplementary file 1 — Supplement 1. Full ITT analysis. [file EPH-111-2975-s002.pdf]

Supplement 1: Full ITT analysis

	Estimated means			Mean differences			
	Baseline	Follow-up: Control	Follow-up: Intervention	Within-group differences: Control	Within-group differences: Intervention	Between-group differences	N
**Weight (kg)**	89.71 [79.83 to 99.59]	90.23 [80.26 to 100.20]	93.84 [83.89 to 103.79]	0.52 [-1.31 to 2.35]; P=0.571	4.13 [2.47 to 5.78]; P<0.0001	3.61 [1.14 to 6.07]; P=0.00503	EX:14, CON:14
**BMI (kg/m2)**	30.40 [27.13 to 33.66]	30.51 [27.22 to 33.80]	31.66 [28.37 to 34.95]	0.12 [-0.48 to 0.71]; P=0.697	1.26 [0.73 to 1.80]; P<0.0001	1.15 [0.35 to 1.95]; P=0.00589	EX:14, CON:14
**Total fat (g)**	33560.42 [27649.75 to 39471.08]	33344.33 [27374.05 to 39314.61]	34309.94 [28347.16 to 40272.71]	-216.09 [-1423.76 to 991.58]; P=0.720	749.52 [-383.35 to 1882.38]; P=0.190	965.60 [-688.69 to 2619.89]; P=0.246	EX:14, CON:14
**Total fat (%)**	38.71 [35.83 to 41.58]	38.46 [35.51 to 41.40]	38.17 [35.23 to 41.10]	-0.25 [-1.16 to 0.67]; P=0.586	-0.54 [-1.40 to 0.32]; P=0.215	-0.29 [-1.54 to 0.96]; P=0.647	EX:14, CON:14
**Android fat mass (g)**	3403.96 [2783.07 to 4024.86]	3414.12 [2778.32 to 4049.91]	3546.28 [2912.37 to 4180.20]	10.15 [-185.26 to 205.57]; P=0.917	142.32 [-40.98 to 325.62]; P=0.125	132.17 [-135.16 to 399.49]; P=0.325	EX:14, CON:14
**Android fat mass (%)**	47.15 [44.01 to 50.29]	47.21 [43.94 to 50.47]	47.23 [43.98 to 50.47]	0.05 [-1.21 to 1.31]; P=0.934	0.07 [-1.11 to 1.25]; P=0.900	0.02 [-1.70 to 1.74]; P=0.980	EX:14, CON:14
**Gynoid fat mass (g)**	4484.00 [3847.24 to 5120.77]	4459.88 [3799.51 to 5120.25]	4678.43 [4021.04 to 5335.81]	-24.13 [-273.05 to 224.80]; P=0.846	194.42 [-39.07 to 427.91]; P=0.101	218.55 [-121.55 to 558.65]; P=0.202	EX:14, CON:14
**Gynoid fat mass (%)**	41.75 [38.89 to 44.61]	41.17 [38.19 to 44.16]	40.72 [37.75 to 43.68]	-0.57 [-1.75 to 0.60]; P=0.331	-1.03 [-2.14 to 0.07]; P=0.0660	-0.46 [-2.06 to 1.15]; P=0.569	EX:14, CON:14
**Total muscle mass (g)**	51842.61 [47978.63 to 55706.58]	51922.99 [47972.42 to 55873.57]	54420.54 [50480.89 to 58360.19]	80.39 [-1095.06 to 1255.83]; P=0.891	2577.93 [1475.33 to 3680.53]; P<0.0001	2497.54 [889.29 to 4105.80]; P=0.00305	EX:14, CON:14

**Bone mass Density (g/cm3)**	1.23 [1.19 to 1.26]	1.22 [1.18 to 1.26]	1.23 [1.19 to 1.27]	-0.01 [-0.02 to 0.01]; P=0.317	0.00 [-0.01 to 0.01]; P=0.934	0.01 [-0.01 to 0.03]; P=0.430	EX:14, CON:14
**FEV1 (L)**	3.10 [2.75 to 3.45]	3.30 [2.91 to 3.69]	3.34 [2.95 to 3.73]	0.20 [-0.05 to 0.45]; P=0.111	0.24 [0.01 to 0.48]; P=0.0418	0.04 [-0.30 to 0.38]; P=0.805	EX:14, CON:14
**FEV1 (% predicted)**	99.19 [93.20 to 105.18]	110.93 [103.31 to 118.56]	111.31 [103.89 to 118.72]	11.74 [5.20 to 18.28]; P=0.000697	12.12 [5.99 to 18.24]; P=0.000222	0.37 [-8.31 to 9.05]; P=0.932	EX:14, CON:14
**FVC (L)**	3.87 [3.45 to 4.29]	4.25 [3.78 to 4.71]	4.19 [3.73 to 4.65]	0.37 [0.10 to 0.65]; P=0.00913	0.32 [0.06 to 0.58]; P=0.0165	-0.05 [-0.43 to 0.32]; P=0.777	EX:14, CON:14
**FVC (% predicted)**	98.55 [92.59 to 104.51]	111.54 [104.07 to 119.02]	109.16 [101.88 to 116.45]	12.99 [6.74 to 19.25]; P=0.000117	10.61 [4.75 to 16.48]; P=0.000646	-2.38 [-10.71 to 5.95]; P=0.570	EX:14, CON:14
**FEV1/FVC (L)**	80.01 [77.89 to 82.12]	79.24 [76.61 to 81.88]	79.99 [77.42 to 82.55]	-0.77 [-2.94 to 1.41]; P=0.483	-0.02 [-2.06 to 2.01]; P=0.981	0.74 [-2.16 to 3.64]; P=0.610	EX:14, CON:14
**FEV1/FVC (% predicted)**	101.50 [98.96 to 104.04]	100.42 [97.15 to 103.69]	102.29 [99.11 to 105.46]	-1.08 [-3.94 to 1.78]; P=0.451	0.78 [-1.90 to 3.47]; P=0.560	1.87 [-1.92 to 5.66]; P=0.328	EX:14, CON:14
**TLC (L)**	5.32 [4.82 to 5.83]	5.99 [5.40 to 6.57]	6.08 [5.51 to 6.65]	0.66 [0.26 to 1.06]; P=0.00159	0.76 [0.38 to 1.13]; P=0.000167	0.10 [-0.44 to 0.63]; P=0.724	EX:14, CON:14
**TLC (% predicted)**	87.42 [81.82 to 93.01]	99.37 [92.34 to 106.40]	97.37 [90.53 to 104.22]	11.96 [6.05 to 17.86]; P=0.000164	9.96 [4.42 to 15.50]; P=0.000690	-2.00 [-9.86 to 5.86]; P=0.613	EX:14, CON:14
**RV (L)**	1.95 [1.75 to 2.15]	2.16 [1.89 to 2.42]	2.11 [1.85 to 2.37]	0.21 [-0.03 to 0.45]; P=0.0917	0.16 [-0.06 to 0.39]; P=0.158	-0.05 [-0.36 to 0.27]; P=0.777	EX:14, CON:14

**RV(% predicted)**	87.39 [79.70 to 95.08]	97.65 [87.03 to 108.28]	93.53 [83.30 to 103.76]	10.26 [0.05 to 20.47]; P=0.0489	6.14 [-3.43 to 15.71]; P=0.204	-4.12 [-17.41 to 9.16]; P=0.537	EX:14, CON:14
**DLCOc (mmol/(min*kPA))**	7.00 [6.21 to 7.80]	7.75 [6.86 to 8.64]	7.70 [6.82 to 8.59]	0.75 [0.17 to 1.32]; P=0.0124	0.70 [0.16 to 1.24]; P=0.0116	-0.04 [-0.82 to 0.73]; P=0.909	EX:14, CON:14
**DLCOc (% predicted)**	78.78 [72.67 to 84.89]	88.09 [80.37 to 95.80]	85.63 [78.11 to 93.15]	9.31 [2.66 to 15.96]; P=0.00698	6.85 [0.65 to 13.05]; P=0.0309	-2.45 [-11.25 to 6.34]; P=0.578	EX:14, CON:14
**VA (L)**	5.30 [4.85 to 5.76]	5.72 [5.20 to 6.24]	5.77 [5.25 to 6.28]	0.42 [0.06 to 0.77]; P=0.0230	0.46 [0.13 to 0.79]; P=0.00739	0.05 [-0.43 to 0.52]; P=0.850	EX:14, CON:14
**VA (% predicted)**	88.84 [83.86 to 93.82]	95.81 [89.73 to 101.89]	94.93 [88.98 to 100.88]	6.97 [2.03 to 11.91]; P=0.00658	6.09 [1.49 to 10.69]; P=0.0105	-0.88 [-7.45 to 5.69]; P=0.790	EX:14, CON:14
**KCOc (mmol/(min*kPA*L))**	1.31 [1.20 to 1.42]	1.36 [1.24 to 1.48]	1.34 [1.23 to 1.46]	0.05 [-0.02 to 0.11]; P=0.138	0.03 [-0.03 to 0.09]; P=0.289	-0.02 [-0.10 to 0.07]; P=0.708	EX:14, CON:14
**KCOc (% predicted)**	89.49 [82.50 to 96.48]	92.99 [85.34 to 100.63]	92.68 [85.11 to 100.25]	3.50 [-0.95 to 7.94]; P=0.120	3.19 [-0.95 to 7.32]; P=0.128	-0.31 [-6.32 to 5.70]; P=0.918	EX:14, CON:14
**Absolute VO2max (L/min)**	1909.33 [1643.02 to 2175.64]	2087.55 [1759.28 to 2415.82]	2284.82 [1984.96 to 2584.67]	178.22 [-62.60 to 419.04]; P=0.143	375.49 [181.16 to 569.83]; P=0.000336	197.27 [-108.30 to 502.84]; P=0.200	EX:14, CON:14
**Relative VO2max (mL/kg/min)**	21.63 [19.10 to 24.16]	23.65 [20.37 to 26.93]	24.77 [21.84 to 27.71]	2.02 [-0.59 to 4.63]; P=0.126	3.14 [1.04 to 5.25]; P=0.00436	1.12 [-2.17 to 4.42]; P=0.497	EX:14, CON:14
**Watt max (W)**	153.90 [127.39 to 180.42]	204.62 [167.09 to 242.16]	194.78 [161.39 to 228.18]	50.72 [15.98 to 85.46]; P=0.00505	40.88 [12.11 to 69.65]; P=0.00633	-9.84 [-53.32 to 33.64]; P=0.652	EX:14, CON:14

**Chestpress 1RM (kg)**	41.82 [34.66 to 48.98]	44.24 [36.37 to 52.11]	45.44 [37.59 to 53.29]	2.42 [-2.08 to 6.93]; P=0.284	3.62 [-0.79 to 8.03]; P=0.105	1.20 [-5.05 to 7.45]; P=0.701	EX:13, CON:14
**Legpress 1RM (kg)**	135.48 [116.80 to 154.15]	153.92 [130.28 to 177.55]	145.45 [122.54 to 168.35]	18.44 [-1.31 to 38.19]; P=0.0666	9.97 [-8.42 to 28.36]; P=0.281	-8.47 [-34.78 to 17.84]; P=0.521	EX:13, CON:14
**FAS score (/50)**	26.74 [24.71 to 28.77]	24.50 [21.71 to 27.28]	23.56 [20.89 to 26.24]	-2.24 [-5.21 to 0.73]; P=0.136	-3.17 [-5.92 to -0.43]; P=0.0244	-0.93 [-4.60 to 2.73]; P=0.613	EX:14, CON:14
**Physical functioning (/100)**	74.09 [66.31 to 81.00]	83.94 [72.80 to 93.72]	82.78 [71.69 to 91.44]	9.86 [0.76 to 20.75]	8.69 [-3.30 to 19.05]	-1.17 [-15.82 to 12.57]	EX:14, CON:14
**Physical role limitations (/100)**	52.70 [39.04 to 69.87]	62.01 [39.85 to 80.97]	81.69 [60.12 to 101.78]	9.32 [-9.04 to 27.33]	28.99 [3.32 to 54.29]	19.68 [-10.89 to 49.56]	EX:14, CON:14
**Emotional role limitations (/100)**	69.61 [57.32 to 83.15]	82.73 [71.11 to 96.75]	92.91 [79.16 to 107.98]	13.12 [1.01 to 31.36]	23.30 [7.29 to 43.58]	10.18 [-9.84 to 31.28]	EX:14, CON:14
**Energy/fatigue (/100)**	52.86 [43.85 to 61.87]	59.53 [48.64 to 70.43]	65.04 [54.39 to 75.69]	6.68 [-1.83 to 15.18]; P=0.121	12.18 [4.21 to 20.15]; P=0.00344	5.51 [-5.89 to 16.90]; P=0.337	EX:14, CON:14
**Emotional well-being (/100)**	78.53 [72.62 to 84.45]	81.97 [74.31 to 89.63]	81.00 [73.57 to 88.43]	3.44 [-3.30 to 10.18]; P=0.311	2.47 [-3.86 to 8.79]; P=0.438	-0.97 [-9.89 to 7.95]; P=0.828	EX:14, CON:14
**Social functioning (/100)**	75.80 [65.52 to 84.82]	84.52 [73.39 to 98.03]	95.73 [86.03 to 103.88]	8.72 [-1.66 to 26.63]	19.92 [9.17 to 32.42]	11.21 [-8.65 to 24.27]	EX:14, CON:14
**Pain (/100)**	73.87 [64.21 to 84.04]	73.58 [61.01 to 87.89]	75.02 [63.85 to 87.63]	-0.29 [-12.46 to 18.26]	1.16 [-7.85 to 13.90]	1.45 [-15.24 to 17.60]	EX:14, CON:14

**General health (/100)**	62.96 [55.43 to 70.49]	60.03 [49.39 to 70.67]	65.45 [55.23 to 75.67]	-2.93 [-13.44 to 7.58]; P=0.579	2.49 [-7.36 to 12.34]; P=0.614	5.42 [-8.15 to 18.99]; P=0.428	EX:14, CON:14
**LVESV (mL)**	49.13 [43.12 to 55.15]	39.30 [31.50 to 47.10]	47.00 [39.55 to 54.46]	-9.83 [-16.60 to -3.06]; P=0.00530	-2.13 [-8.40 to 4.14]; P=0.498	7.70 [-1.21 to 16.62]; P=0.0891	EX:13, CON:13
**LVEDV (mL)**	133.06 [121.55 to 144.58]	124.75 [111.18 to 138.32]	136.60 [123.43 to 149.77]	-8.31 [-18.15 to 1.52]; P=0.0957	3.54 [-5.56 to 12.63]; P=0.438	11.85 [-1.31 to 25.00]; P=0.0765	EX:13, CON:13
**LVSV (mL)**	83.94 [75.86 to 92.02]	85.55 [75.64 to 95.46]	89.46 [79.91 to 99.01]	1.61 [-6.22 to 9.44]; P=0.681	5.52 [-1.73 to 12.76]; P=0.132	3.91 [-6.50 to 14.32]; P=0.455	EX:13, CON:13
**EF (%)**	63.00 [60.30 to 65.71]	68.50 [64.68 to 72.32]	65.00 [61.39 to 68.60]	5.50 [1.80 to 9.19]; P=0.00429	1.99 [-1.43 to 5.42]; P=0.248	-3.50 [-8.27 to 1.26]; P=0.146	EX:13, CON:13
**CO (L/min)**	6.01 [5.41 to 6.61]	6.24 [5.43 to 7.04]	5.98 [5.21 to 6.74]	0.23 [-0.51 to 0.97]; P=0.544	-0.03 [-0.72 to 0.65]; P=0.922	-0.26 [-1.22 to 0.71]; P=0.594	EX:13, CON:13
**CO/BSA (L/min/m2)**	2.95 [2.75 to 3.16]	3.10 [2.79 to 3.41]	2.90 [2.61 to 3.19]	0.14 [-0.18 to 0.46]; P=0.380	-0.05 [-0.35 to 0.25]; P=0.732	-0.19 [-0.60 to 0.21]; P=0.343	EX:13, CON:13
**PER (mL/s)**	506.55 [462.62 to 550.47]	547.73 [482.89 to 612.56]	446.79 [386.01 to 507.58]	41.18 [-24.74 to 107.11]; P=0.216	-59.75 [-120.98 to 1.47]; P=0.0556	-100.93 [-184.48 to -17.39]; P=0.0187	EX:13, CON:13
**PFR (mL/s)**	421.97 [371.86 to 472.08]	412.41 [341.92 to 482.91]	415.29 [348.72 to 481.86]	-9.56 [-77.46 to 58.34]; P=0.779	-6.68 [-69.65 to 56.28]; P=0.832	2.87 [-84.74 to 90.49]; P=0.948	EX:13, CON:13
**LVESV/BSA (mL/m2)**	24.25 [21.71 to 26.78]	19.50 [16.06 to 22.94]	22.87 [19.61 to 26.14]	-4.74 [-7.92 to -1.57]; P=0.00416	-1.37 [-4.31 to 1.57]; P=0.353	3.37 [-0.77 to 7.51]; P=0.108	EX:13, CON:13

**LVESV/H (mL/m)**	28.25 [25.28 to 31.22]	22.63 [18.60 to 26.66]	26.93 [23.10 to 30.75]	-5.62 [-9.34 to -1.90]; P=0.00377	-1.32 [-4.77 to 2.12]; P=0.444	4.30 [-0.55 to 9.14]; P=0.0811	EX:13, CON:13
**LVEDV/BSA (mL/m2)**	65.44 [61.12 to 69.75]	61.63 [56.24 to 67.01]	65.93 [60.75 to 71.11]	-3.81 [-8.20 to 0.58]; P=0.0874	0.49 [-3.57 to 4.55]; P=0.809	4.30 [-1.52 to 10.12]; P=0.144	EX:13, CON:13
**LVEDV/H (mL/m)**	76.50 [71.18 to 81.81]	72.00 [65.36 to 78.65]	78.18 [71.79 to 84.57]	-4.49 [-9.93 to 0.95]; P=0.103	1.68 [-3.35 to 6.71]; P=0.506	6.17 [-1.04 to 13.38]; P=0.0919	EX:13, CON:13
**LVSV/BSA (mL/m2)**	41.21 [38.04 to 44.38]	42.19 [38.13 to 46.25]	43.12 [39.24 to 47.01]	0.98 [-2.48 to 4.43]; P=0.572	1.92 [-1.28 to 5.12]; P=0.234	0.94 [-3.62 to 5.50]; P=0.682	EX:13, CON:13
**LVSV/H (mL/m)**	48.26 [44.21 to 52.31]	49.40 [44.22 to 54.58]	51.21 [46.25 to 56.17]	1.14 [-3.26 to 5.55]; P=0.604	2.95 [-1.13 to 7.02]; P=0.152	1.80 [-4.01 to 7.62]; P=0.536	EX:13, CON:13
**LVMASS (g)**	114.54 [105.16 to 123.92]	104.93 [94.63 to 115.24]	114.61 [104.49 to 124.73]	-9.61 [-15.51 to -3.71]; P=0.00201	0.07 [-5.38 to 5.52]; P=0.979	9.68 [1.72 to 17.64]; P=0.0182	EX:13, CON:13
**LVMASS/BSA (g/m2)**	55.70 [52.51 to 58.89]	52.10 [48.15 to 56.05]	54.61 [50.81 to 58.42]	-3.60 [-6.78 to -0.42]; P=0.0273	-1.09 [-4.03 to 1.85]; P=0.460	2.51 [-1.71 to 6.73]; P=0.238	EX:13, CON:13
**LVMASS/H (g/m)**	65.79 [60.61 to 70.97]	61.78 [55.88 to 67.68]	65.52 [59.76 to 71.27]	-4.01 [-7.88 to -0.14]; P=0.0425	-0.27 [-3.85 to 3.30]; P=0.879	3.74 [-1.46 to 8.93]; P=0.155	EX:13, CON:13
**T1 global (msec)**	1024.35 [1013.60 to 1035.11]	1017.66 [1001.14 to 1034.19]	1012.41 [997.07 to 1027.76]	-6.69 [-23.53 to 10.15]; P=0.429	-11.94 [-27.43 to 3.55]; P=0.128	-5.25 [-26.63 to 16.14]; P=0.625	EX:13, CON:13
**T2 global (msec)**	47.96 [47.40 to 48.62]	47.64 [46.13 to 48.72]	47.92 [47.28 to 49.04]	-0.32 [-2.12 to 0.79]	-0.04 [-0.82 to 0.82]	0.28 [-0.96 to 2.09]	EX:13, CON:13

**Psychological (/100)**	74.41 [68.91 to 80.69]	88.28 [79.05 to 95.67]	89.88 [82.15 to 99.25]	13.87 [5.30 to 20.78]	15.47 [5.20 to 27.13]	1.60 [-9.51 to 15.72]	EX:14, CON:14
**Breathlessness & activities (/100)**	47.03 [40.11 to 54.49]	67.18 [57.77 to 77.49]	66.34 [57.75 to 79.43]	20.14 [11.79 to 28.85]	19.30 [11.62 to 33.57]	-0.84 [-11.69 to 14.79]	EX:14, CON:14
**Chest symptoms (/100)**	78.88 [72.36 to 84.25]	91.93 [87.10 to 99.72]	91.21 [79.29 to 99.33]	13.04 [8.18 to 22.26]	12.32 [-3.09 to 22.17]	-0.72 [-18.43 to 9.25]	EX:14, CON:14
**Total score (/100)**	64.37 [59.10 to 69.65]	78.68 [71.78 to 85.58]	77.45 [70.77 to 84.14]	14.30 [8.13 to 20.48]; P<0.0001	13.08 [7.29 to 18.86]; P<0.0001	-1.23 [-9.37 to 6.92]; P=0.765	EX:14, CON:14
