# Supplementary material for: Impact of a 12‐week high‐intensity interval training intervention on cardiac structure and function after COVID‐19 at 12‐month follow‐up
Source: Exp Physiol. 2024 Sep 11;111(6):2975–86. doi: 10.1113/EP092099 (PMC13238539; doi:10.1113/EP092099)
Supplement: Supplementary file 2 — Supplement 2. PP analysis. [file EPH-111-2975-s001.pdf]

Supplement 2: PP analysis

	Estimated means			Mean differences			
	Baseline	Follow-up: Control	Follow-up: Intervention	Within-group differences: Control	Within-group differences: Intervention	Between-group differences	N
**Weight (kg)**	80.12 [73.63 to 86.61]	80.85 [74.24 to 87.45]	84.81 [78.22 to 91.41]	0.73 [-1.17 to 2.62]; P=0.443	4.69 [2.86 to 6.52]; P<0.0001	3.97 [1.34 to 6.60]; P=0.00405	EX:11, CON:11
**BMI (kg/m2)**	27.11 [25.38 to 28.85]	27.27 [25.49 to 29.05]	28.56 [26.79 to 30.34]	0.16 [-0.45 to 0.78]; P=0.599	1.45 [0.86 to 2.05]; P<0.0001	1.29 [0.44 to 2.14]; P=0.00380	EX:11, CON:11
**Total fat (g)**	28211.73 [23817.01 to 32606.46]	28108.39 [23640.87 to 32575.90]	28993.26 [24519.97 to 33466.55]	-103.35 [-1389.80 to 1183.11]; P=0.872	781.53 [-504.93 to 2067.99]; P=0.227	884.88 [-929.50 to 2699.26]; P=0.330	EX:11, CON:11
**Total fat (%)**	36.78 [33.80 to 39.77]	36.48 [33.43 to 39.53]	36.20 [33.15 to 39.25]	-0.30 [-1.29 to 0.68]; P=0.537	-0.59 [-1.58 to 0.40]; P=0.238	-0.28 [-1.68 to 1.11]; P=0.686	EX:11, CON:11
**Android fat mass (g)**	2990.13 [2440.43 to 3539.82]	2978.89 [2417.35 to 3540.42]	3157.09 [2594.61 to 3719.57]	-11.24 [-194.46 to 171.98]; P=0.902	166.97 [-16.26 to 350.19]; P=0.0730	178.21 [-79.99 to 436.41]; P=0.171	EX:11, CON:11
**Android fat mass (%)**	45.17 [42.03 to 48.30]	45.06 [41.82 to 48.30]	45.34 [42.09 to 48.59]	-0.11 [-1.41 to 1.19]; P=0.865	0.17 [-1.12 to 1.47]; P=0.788	0.28 [-1.54 to 2.11]; P=0.755	EX:11, CON:11
**Gynoid fat mass (g)**	4583.88 [3941.62 to 5226.14]	4607.46 [3950.13 to 5264.80]	4671.66 [4013.13 to 5330.19]	23.58 [-199.67 to 246.84]; P=0.832	87.78 [-135.47 to 311.03]; P=0.432	64.19 [-250.31 to 378.70]; P=0.682	EX:11, CON:11
**Gynoid fat mass (%)**	40.32 [37.36 to 43.28]	39.92 [36.88 to 42.96]	38.79 [35.74 to 41.83]	-0.40 [-1.51 to 0.72]; P=0.477	-1.53 [-2.65 to -0.42]; P=0.00828	-1.14 [-2.70 to 0.43]; P=0.151	EX:11, CON:11
**Total muscle mass (g)**	48817.85 [45692.85 to 51942.84]	49162.19 [45948.94 to 52375.45]	51611.77 [48391.53 to 54832.02]	344.35 [-845.87 to 1534.56]; P=0.562	2793.93 [1603.71 to 3984.14]; P<0.0001	2449.58 [774.18 to 4124.99]; P=0.00517	EX:11, CON:11

**Bone mass Density (g/cm3)**	1.20 [1.16 to 1.24]	1.20 [1.16 to 1.24]	1.20 [1.16 to 1.24]	-0.01 [-0.02 to 0.01]; P=0.399	-0.00 [-0.01 to 0.01]; P=0.878	0.00 [-0.01 to 0.02]; P=0.623	EX:11, CON:11
**FEV1 (L)**	3.07 [2.64 to 3.50]	3.28 [2.83 to 3.74]	3.40 [2.94 to 3.86]	0.21 [-0.03 to 0.45]; P=0.0883	0.33 [0.08 to 0.57]; P=0.00924	0.12 [-0.22 to 0.45]; P=0.486	EX:11, CON:11
**FEV1 (% predicted)**	100.23 [92.76 to 107.69]	112.11 [103.39 to 120.82]	112.72 [103.91 to 121.52]	11.88 [4.94 to 18.82]; P=0.00124	12.49 [5.55 to 19.43]; P=0.000735	0.61 [-8.90 to 10.12]; P=0.898	EX:11, CON:11
**FVC (L)**	3.91 [3.39 to 4.42]	4.29 [3.74 to 4.84]	4.22 [3.67 to 4.77]	0.39 [0.09 to 0.68]; P=0.0109	0.31 [0.02 to 0.61]; P=0.0369	-0.07 [-0.49 to 0.34]; P=0.718	EX:11, CON:11
**FVC (% predicted)**	99.72 [92.35 to 107.09]	113.08 [104.53 to 121.64]	110.72 [102.08 to 119.37]	13.36 [6.66 to 20.06]; P=0.000223	11.00 [4.30 to 17.70]; P=0.00186	-2.36 [-11.55 to 6.84]; P=0.609	EX:11, CON:11
**FEV1/FVC (L)**	79.42 [76.94 to 81.89]	78.56 [75.69 to 81.42]	79.58 [76.68 to 82.47]	-0.86 [-3.10 to 1.38]; P=0.443	0.16 [-2.07 to 2.40]; P=0.883	1.02 [-2.05 to 4.10]; P=0.507	EX:11, CON:11
**FEV1/FVC (% predicted)**	100.83 [97.76 to 103.89]	99.71 [96.07 to 103.34]	101.95 [98.28 to 105.62]	-1.12 [-4.13 to 1.88]; P=0.456	1.12 [-1.88 to 4.13]; P=0.456	2.24 [-1.86 to 6.34]; P=0.277	EX:11, CON:11
**TLC (L)**	5.40 [4.81 to 5.99]	6.11 [5.46 to 6.75]	6.06 [5.41 to 6.71]	0.71 [0.30 to 1.12]; P=0.00115	0.66 [0.25 to 1.07]; P=0.00231	-0.05 [-0.62 to 0.52]; P=0.862	EX:11, CON:11
**TLC (% predicted)**	88.98 [82.50 to 95.45]	101.44 [93.90 to 108.97]	97.69 [90.08 to 105.31]	12.46 [6.51 to 18.41]; P=0.000118	8.72 [2.76 to 14.67]; P=0.00503	-3.75 [-11.91 to 4.42]; P=0.361	EX:11, CON:11
**RV (L)**	1.95 [1.73 to 2.17]	2.16 [1.89 to 2.43]	2.08 [1.80 to 2.35]	0.21 [-0.03 to 0.46]; P=0.0883	0.13 [-0.12 to 0.37]; P=0.300	-0.08 [-0.41 to 0.25]; P=0.610	EX:11, CON:11

**RV(% predicted)**	86.20 [77.17 to 95.23]	97.31 [86.02 to 108.60]	91.27 [79.82 to 102.71]	11.11 [0.60 to 21.63]; P=0.0388	5.07 [-5.44 to 15.58]; P=0.337	-6.04 [-20.12 to 8.04]; P=0.393	EX:11, CON:11
**DLCOc (mmol/(min*kPA))**	6.92 [5.96 to 7.88]	7.67 [6.64 to 8.70]	7.51 [6.48 to 8.55]	0.75 [0.15 to 1.34]; P=0.0151	0.59 [-0.00 to 1.19]; P=0.0504	-0.15 [-0.99 to 0.68]; P=0.712	EX:11, CON:11
**DLCOc (% predicted)**	77.63 [70.45 to 84.82]	87.09 [78.64 to 95.55]	83.79 [75.25 to 92.34]	9.46 [2.59 to 16.32]; P=0.00803	6.16 [-0.71 to 13.03]; P=0.0775	-3.30 [-12.69 to 6.09]; P=0.484	EX:11, CON:11
**VA (L)**	5.37 [4.81 to 5.93]	5.76 [5.16 to 6.36]	5.66 [5.05 to 6.26]	0.39 [0.04 to 0.74]; P=0.0299	0.29 [-0.06 to 0.64]; P=0.104	-0.10 [-0.59 to 0.39]; P=0.678	EX:11, CON:11
**VA (% predicted)**	90.29 [84.09 to 96.48]	96.68 [89.72 to 103.64]	94.42 [87.40 to 101.45]	6.39 [1.47 to 11.32]; P=0.0122	4.13 [-0.79 to 9.06]; P=0.0978	-2.26 [-9.07 to 4.56]; P=0.509	EX:11, CON:11
**KCOc (mmol/(min*kPA*L))**	1.28 [1.15 to 1.41]	1.34 [1.20 to 1.47]	1.33 [1.19 to 1.47]	0.05 [-0.02 to 0.12]; P=0.124	0.05 [-0.02 to 0.12]; P=0.169	-0.01 [-0.10 to 0.09]; P=0.903	EX:11, CON:11
**KCOc (% predicted)**	87.99 [79.48 to 96.50]	91.82 [82.81 to 100.82]	92.29 [83.25 to 101.34]	3.83 [-0.80 to 8.46]; P=0.103	4.30 [-0.33 to 8.93]; P=0.0678	0.47 [-6.01 to 6.96]; P=0.883	EX:11, CON:11
**Absolute VO2max (L/min)**	1827.78 [1527.34 to 2128.22]	2024.52 [1672.90 to 2376.14]	2242.32 [1908.70 to 2575.94]	196.74 [-51.41 to 444.89]; P=0.117	414.54 [197.28 to 631.80]; P=0.000418	217.80 [-106.94 to 542.54]; P=0.183	EX:11, CON:11
**Relative VO2max (mL/kg/min)**	22.77 [19.78 to 25.76]	24.62 [21.03 to 28.22]	26.00 [22.62 to 29.38]	1.85 [-0.84 to 4.55]; P=0.172	3.23 [0.87 to 5.59]; P=0.00850	1.38 [-2.14 to 4.89]; P=0.433	EX:11, CON:11
**Watt max (W)**	158.65 [127.93 to 189.37]	208.38 [168.66 to 248.10]	202.98 [165.41 to 240.54]	49.73 [13.38 to 86.08]; P=0.00846	44.32 [11.82 to 76.82]; P=0.00872	-5.40 [-52.07 to 41.26]; P=0.817	EX:11, CON:11

**Chestpress 1RM (kg)**	41.33 [32.96 to 49.70]	43.49 [34.57 to 52.41]	44.56 [35.61 to 53.52]	2.16 [-2.46 to 6.77]; P=0.350	3.23 [-1.43 to 7.89]; P=0.168	1.07 [-5.43 to 7.58]; P=0.740	EX:11, CON:11
**Legpress 1RM (kg)**	133.85 [114.23 to 153.47]	155.11 [131.08 to 179.14]	142.24 [118.57 to 165.91]	21.25 [0.66 to 41.85]; P=0.0434	8.39 [-11.30 to 28.07]; P=0.395	-12.87 [-40.37 to 14.63]; P=0.351	EX:11, CON:11
**FAS score (/50)**	26.19 [23.92 to 28.46]	24.05 [21.27 to 26.83]	22.32 [19.49 to 25.16]	-2.14 [-5.24 to 0.96]; P=0.172	-3.86 [-6.92 to -0.81]; P=0.0144	-1.73 [-5.53 to 2.08]; P=0.367	EX:11, CON:11
**Physical functioning (/100)**	76.56 [69.50 to 82.50]	86.37 [77.34 to 95.24]	89.93 [81.97 to 96.14]	9.81 [1.17 to 20.16]	13.37 [4.42 to 23.01]	3.56 [-8.84 to 15.96]	EX:11, CON:11
**Physical role limitations (/100)**	54.65 [38.00 to 70.87]	64.18 [37.92 to 82.91]	86.18 [67.02 to 108.62]	9.54 [-12.16 to 27.13]	31.53 [8.33 to 60.12]	21.99 [-4.46 to 55.50]	EX:11, CON:11
**Emotional role limitations (/100)**	73.96 [61.22 to 86.56]	84.80 [72.20 to 96.94]	96.45 [82.36 to 113.02]	10.84 [-1.51 to 26.51]	22.49 [2.95 to 44.71]	11.65 [-10.21 to 34.91]	EX:11, CON:11
**Energy/fatigue (/100)**	57.23 [48.21 to 66.25]	62.70 [52.06 to 73.34]	71.30 [60.54 to 82.06]	5.47 [-3.23 to 14.18]; P=0.212	14.07 [5.37 to 22.77]; P=0.00214	8.60 [-3.29 to 20.49]; P=0.153	EX:11, CON:11
**Emotional well-being (/100)**	78.90 [72.59 to 85.22]	81.92 [74.16 to 89.68]	82.43 [74.58 to 90.29]	3.02 [-3.93 to 9.97]; P=0.387	3.53 [-3.42 to 10.48]; P=0.312	0.51 [-8.87 to 9.89]; P=0.914	EX:11, CON:11
**Social functioning (/100)**	77.15 [66.18 to 85.96]	86.68 [77.88 to 100.01]	95.81 [86.98 to 104.55]	9.53 [-1.40 to 30.87]	18.65 [6.76 to 31.52]	9.13 [-11.05 to 23.41]	EX:11, CON:11
**Pain (/100)**	72.92 [61.24 to 82.68]	73.83 [61.40 to 89.49]	77.01 [66.86 to 88.85]	0.91 [-11.46 to 22.20]	4.09 [-2.21 to 19.37]	3.18 [-14.84 to 19.06]	EX:11, CON:11

**General health (/100)**	63.94 [55.32 to 72.55]	60.01 [49.06 to 70.97]	69.65 [58.54 to 80.75]	-3.93 [-14.45 to 6.60]; P=0.457	5.71 [-4.82 to 16.23]; P=0.281	9.63 [-4.36 to 23.62]; P=0.173	EX:11, CON:11
**LVESV (mL)**	48.54 [42.29 to 54.79]	39.42 [31.63 to 47.21]	45.40 [37.65 to 53.16]	-9.12 [-15.77 to -2.46]; P=0.00838	-3.14 [-9.74 to 3.47]; P=0.344	5.98 [-3.06 to 15.03]; P=0.190	EX:11, CON:11
**LVEDV (mL)**	130.19 [118.57 to 141.81]	122.63 [109.07 to 136.19]	135.70 [122.19 to 149.21]	-7.56 [-17.61 to 2.49]; P=0.136	5.51 [-4.45 to 15.48]; P=0.271	13.07 [-0.77 to 26.92]; P=0.0636	EX:11, CON:11
**LVSV (mL)**	81.67 [73.07 to 90.27]	83.35 [73.13 to 93.57]	90.16 [79.98 to 100.34]	1.68 [-6.25 to 9.61]; P=0.671	8.49 [0.63 to 16.36]; P=0.0350	6.81 [-4.07 to 17.70]; P=0.214	EX:11, CON:11
**EF (%)**	62.59 [59.51 to 65.67]	67.90 [63.91 to 71.89]	66.05 [62.07 to 70.02]	5.31 [1.66 to 8.96]; P=0.00532	3.46 [-0.17 to 7.09]; P=0.0610	-1.85 [-6.76 to 3.06]; P=0.453	EX:11, CON:11
**CO (L/min)**	5.89 [5.27 to 6.51]	6.06 [5.26 to 6.86]	5.84 [5.05 to 6.64]	0.17 [-0.56 to 0.89]; P=0.643	-0.05 [-0.77 to 0.68]; P=0.899	-0.21 [-1.19 to 0.76]; P=0.662	EX:11, CON:11
**CO/BSA (L/min/m2)**	2.99 [2.76 to 3.22]	3.10 [2.78 to 3.42]	2.92 [2.60 to 3.24]	0.11 [-0.22 to 0.44]; P=0.508	-0.06 [-0.39 to 0.26]; P=0.700	-0.17 [-0.60 to 0.26]; P=0.425	EX:11, CON:11
**PER (mL/s)**	506.91 [459.44 to 554.37]	543.45 [478.39 to 608.50]	464.06 [399.20 to 528.92]	36.54 [-28.28 to 101.37]; P=0.262	-42.85 [-107.42 to 21.73]; P=0.188	-79.39 [-164.71 to 5.92]; P=0.0675	EX:11, CON:11
**PFR (mL/s)**	424.20 [368.75 to 479.65]	415.59 [342.00 to 489.18]	389.83 [316.51 to 463.15]	-8.61 [-78.46 to 61.24]; P=0.805	-34.37 [-103.86 to 35.12]; P=0.324	-25.76 [-118.95 to 67.43]; P=0.582	EX:11, CON:11
**LVESV/BSA (mL/m2)**	24.77 [22.00 to 27.54]	20.16 [16.61 to 23.70]	22.69 [19.16 to 26.22]	-4.61 [-7.79 to -1.44]; P=0.00543	-2.08 [-5.24 to 1.08]; P=0.191	2.53 [-1.76 to 6.82]; P=0.241	EX:11, CON:11

**LVESV/H (mL/m)**	28.06 [24.86 to 31.26]	22.78 [18.68 to 26.88]	26.19 [22.10 to 30.27]	-5.28 [-8.98 to -1.59]; P=0.00611	-1.88 [-5.55 to 1.80]; P=0.309	3.41 [-1.58 to 8.39]; P=0.176	EX:11, CON:11
**LVEDV/BSA (mL/m2)**	66.25 [61.53 to 70.97]	62.54 [56.86 to 68.23]	67.34 [61.68 to 73.00]	-3.71 [-8.25 to 0.84]; P=0.107	1.09 [-3.42 to 5.59]; P=0.629	4.80 [-1.43 to 11.02]; P=0.128	EX:11, CON:11
**LVEDV/H (mL/m)**	75.24 [69.65 to 80.82]	71.09 [64.28 to 77.89]	78.17 [71.39 to 84.95]	-4.15 [-9.73 to 1.43]; P=0.141	2.93 [-2.61 to 8.47]; P=0.292	7.08 [-0.54 to 14.70]; P=0.0678	EX:11, CON:11
**LVSV/BSA (mL/m2)**	41.49 [37.88 to 45.10]	42.39 [38.01 to 46.76]	44.67 [40.31 to 49.03]	0.89 [-2.65 to 4.44]; P=0.614	3.18 [-0.34 to 6.69]; P=0.0754	2.28 [-2.56 to 7.13]; P=0.348	EX:11, CON:11
**LVSV/H (mL/m)**	47.19 [42.75 to 51.62]	48.31 [42.90 to 53.72]	51.99 [46.59 to 57.38]	1.12 [-3.33 to 5.57]; P=0.613	4.80 [0.38 to 9.21]; P=0.0340	3.68 [-2.40 to 9.75]; P=0.230	EX:11, CON:11
**LVMASS (g)**	109.39 [100.14 to 118.64]	101.04 [90.99 to 111.10]	109.11 [99.08 to 119.14]	-8.35 [-14.08 to -2.62]; P=0.00540	-0.28 [-5.96 to 5.39]; P=0.920	8.07 [0.09 to 16.05]; P=0.0477	EX:11, CON:11
**LVMASS/BSA (g/m2)**	55.19 [51.67 to 58.71]	51.98 [47.80 to 56.16]	53.69 [49.53 to 57.85]	-3.21 [-6.44 to 0.02]; P=0.0514	-1.50 [-4.70 to 1.70]; P=0.350	1.71 [-2.72 to 6.14]; P=0.442	EX:11, CON:11
**LVMASS/H (g/m)**	63.07 [57.92 to 68.22]	59.76 [53.93 to 65.60]	62.74 [56.92 to 68.55]	-3.31 [-7.26 to 0.65]; P=0.0989	-0.33 [-4.25 to 3.58]; P=0.864	2.97 [-2.50 to 8.45]; P=0.279	EX:11, CON:11
**T1 global (msec)**	1022.76 [1012.23 to 1033.29]	1017.26 [1001.93 to 1032.59]	1005.52 [990.23 to 1020.80]	-5.50 [-21.29 to 10.29]; P=0.486	-17.25 [-32.99 to -1.50]; P=0.0326	-11.75 [-32.45 to 8.96]; P=0.260	EX:11, CON:11
**T2 global (msec)**	48.15 [47.53 to 48.92]	47.63 [46.06 to 48.77]	48.03 [47.17 to 49.06]	-0.52 [-2.35 to 0.69]	-0.12 [-1.14 to 0.85]	0.40 [-1.16 to 2.28]	EX:11, CON:11

**Psychological (/100)**	74.84 [67.71 to 81.91]	88.82 [79.00 to 96.23]	87.26 [79.65 to 97.05]	13.98 [4.05 to 21.44]	12.42 [2.63 to 25.08]	-1.56 [-12.01 to 13.24]	EX:11, CON:11
**Breathlessness & activities (/100)**	49.20 [40.58 to 56.96]	69.38 [58.83 to 79.37]	68.82 [58.96 to 84.80]	20.18 [10.77 to 28.16]	19.62 [10.55 to 35.59]	-0.56 [-12.41 to 17.59]	EX:11, CON:11
**Chest symptoms (/100)**	78.43 [71.67 to 85.42]	92.45 [86.92 to 99.68]	90.43 [77.80 to 99.69]	14.02 [9.10 to 23.89]	12.01 [-4.67 to 22.46]	-2.01 [-19.26 to 9.79]	EX:11, CON:11
**Total score (/100)**	65.07 [58.74 to 71.39]	79.54 [71.95 to 87.14]	77.16 [69.48 to 84.85]	14.48 [7.99 to 20.96]; $P<0.0001$	12.10 [5.62 to 18.58]; $P=0.000485$	-2.38 [-11.19 to 6.43]; $P=0.590$	EX:11, CON:11
