# Supplementary material for: Impact of a 12‐week high‐intensity interval training intervention on cardiac structure and function after COVID‐19 at 12‐month follow‐up
Source: Exp Physiol. 2024 Sep 11;111(6):2975–86. doi: 10.1113/EP092099 (PMC13238539; doi:10.1113/EP092099)
Supplement: Supplementary file 3 — Supplement 3. Sensitivity analysis. [file EPH-111-2975-s003.pdf]

Supplement 3: Sensitivity analysis

	Estimated means			Mean differences			
	Baseline	Follow-up: Control	Follow-up: Intervention	Within-group differences: Control	Within-group differences: Intervention	Between-group differences	N
**Weight (kg)**	79.80 [71.99 to 87.61]	80.79 [72.83 to 88.76]	84.52 [76.65 to 92.39]	0.99 [-1.23 to 3.21]; P=0.370	4.72 [3.05 to 6.39]; P<0.0001	3.73 [0.95 to 6.51]; P=0.0101	EX:11, CON:7
**BMI (kg/m2)**	26.94 [24.84 to 29.05]	27.23 [25.06 to 29.39]	28.41 [26.28 to 30.54]	0.28 [-0.44 to 1.00]; P=0.431	1.46 [0.92 to 2.00]; P<0.0001	1.18 [0.28 to 2.08]; P=0.0116	EX:11, CON:7
**Total fat (g)**	28220.93 [22836.67 to 33605.20]	28297.40 [22818.22 to 33776.57]	29005.27 [23578.12 to 34432.42]	76.47 [-1451.65 to 1604.58]; P=0.919	784.34 [-435.67 to 2004.35]; P=0.200	707.87 [-1244.11 to 2659.85]; P=0.466	EX:11, CON:7
**Total fat (%)**	36.79 [33.10 to 40.48]	36.75 [32.96 to 40.54]	36.20 [32.47 to 39.93]	-0.04 [-1.31 to 1.23]; P=0.949	-0.59 [-1.61 to 0.42]; P=0.243	-0.55 [-2.17 to 1.07]; P=0.494	EX:11, CON:7
**Android fat mass (g)**	2941.53 [2293.49 to 3589.57]	2998.81 [2339.55 to 3658.07]	3110.09 [2456.98 to 3763.19]	57.28 [-125.03 to 239.59]; P=0.527	168.55 [23.00 to 314.10]; P=0.0246	111.27 [-121.61 to 344.16]; P=0.338	EX:11, CON:7
**Android fat mass (%)**	44.69 [41.02 to 48.36]	45.19 [41.35 to 49.03]	44.87 [41.12 to 48.61]	0.50 [-1.19 to 2.18]; P=0.554	0.18 [-1.17 to 1.52]; P=0.793	-0.32 [-2.47 to 1.83]; P=0.764	EX:11, CON:7
**Gynoid fat mass (g)**	4644.68 [3864.20 to 5425.16]	4660.27 [3856.10 to 5464.44]	4731.94 [3940.78 to 5523.11]	15.59 [-273.04 to 304.21]; P=0.913	87.26 [-143.30 to 317.83]; P=0.447	71.68 [-296.63 to 439.98]; P=0.695	EX:11, CON:7
**Gynoid fat mass (%)**	40.74 [37.18 to 44.30]	39.85 [36.15 to 43.54]	39.20 [35.58 to 42.82]	-0.89 [-2.36 to 0.58]; P=0.226	-1.54 [-2.72 to -0.37]; P=0.0118	-0.65 [-2.52 to 1.23]; P=0.487	EX:11, CON:7
**Total muscle mass (g)**	48586.27 [44885.33 to 52287.21]	48653.20 [44807.14 to 52499.26]	51398.59 [47632.24 to 55164.94]	66.93 [-1482.54 to 1616.40]; P=0.930	2812.32 [1574.05 to 4050.59]; P<0.0001	2745.39 [769.54 to 4721.24]; P=0.00791	EX:11, CON:7

**Bone mass Density (g/cm3)**	1.20 [1.15 to 1.25]	1.20 [1.16 to 1.25]	1.20 [1.15 to 1.25]	0.00 [-0.01 to 0.02]; P=0.601	-0.00 [-0.01 to 0.01]; P=0.903	-0.00 [-0.03 to 0.02]; P=0.627	EX:11, CON:7
**FEV1 (L)**	3.10 [2.58 to 3.61]	3.11 [2.56 to 3.66]	3.42 [2.89 to 3.96]	0.02 [-0.27 to 0.30]; P=0.912	0.33 [0.10 to 0.55]; P=0.00589	0.31 [-0.05 to 0.67]; P=0.0869	EX:11, CON:7
**FEV1 (% predicted)**	100.13 [91.67 to 108.59]	108.54 [97.71 to 119.37]	112.69 [103.10 to 122.27]	8.41 [-0.97 to 17.80]; P=0.0775	12.56 [4.96 to 20.15]; P=0.00191	4.15 [-7.55 to 15.84]; P=0.478	EX:11, CON:7
**FVC (L)**	3.93 [3.31 to 4.55]	4.21 [3.54 to 4.89]	4.25 [3.60 to 4.89]	0.28 [-0.11 to 0.68]; P=0.150	0.32 [0.00 to 0.63]; P=0.0490	0.03 [-0.47 to 0.53]; P=0.899	EX:11, CON:7
**FVC (% predicted)**	99.54 [91.35 to 107.73]	111.73 [101.26 to 122.21]	110.78 [101.51 to 120.05]	12.19 [3.11 to 21.27]; P=0.00985	11.24 [3.89 to 18.59]; P=0.00375	-0.95 [-12.27 to 10.36]; P=0.866	EX:11, CON:7
**FEV1/FVC (L)**	79.50 [76.56 to 82.44]	77.14 [73.52 to 80.75]	79.62 [76.36 to 82.87]	-2.36 [-5.29 to 0.57]; P=0.111	0.12 [-2.25 to 2.48]; P=0.921	2.48 [-1.20 to 6.15]; P=0.181	EX:11, CON:7
**FEV1/FVC (% predicted)**	100.98 [97.40 to 104.57]	97.69 [93.18 to 102.20]	101.99 [97.97 to 106.01]	-3.29 [-7.10 to 0.51]; P=0.0877	1.01 [-2.07 to 4.08]; P=0.511	4.30 [-0.45 to 9.05]; P=0.0751	EX:11, CON:7
**TLC (L)**	5.45 [4.76 to 6.14]	6.00 [5.21 to 6.78]	6.11 [5.37 to 6.84]	0.55 [0.01 to 1.08]; P=0.0442	0.66 [0.23 to 1.08]; P=0.00358	0.11 [-0.56 to 0.78]; P=0.740	EX:11, CON:7
**TLC (% predicted)**	90.15 [83.36 to 96.94]	99.70 [90.98 to 108.43]	98.80 [91.10 to 106.50]	9.55 [1.95 to 17.16]; P=0.0152	8.65 [2.49 to 14.81]; P=0.00727	-0.90 [-10.38 to 8.57]; P=0.848	EX:11, CON:7
**RV (L)**	1.98 [1.73 to 2.23]	2.17 [1.82 to 2.52]	2.10 [1.80 to 2.40]	0.19 [-0.14 to 0.52]; P=0.257	0.12 [-0.15 to 0.39]; P=0.379	-0.07 [-0.47 to 0.34]; P=0.731	EX:11, CON:7

**RV(% predicted)**	88.82 [79.12 to 98.52]	96.67 [82.79 to 110.54]	93.35 [81.61 to 105.10]	7.85 [-5.97 to 21.66]; P=0.258	4.53 [-6.80 to 15.87]; P=0.423	-3.31 [-20.11 to 13.49]; P=0.693	EX:11, CON:7
**DLCOc (mmol/(min*kPA))**	7.12 [6.00 to 8.25]	7.79 [6.56 to 9.03]	7.70 [6.52 to 8.87]	0.67 [-0.07 to 1.41]; P=0.0734	0.58 [-0.02 to 1.17]; P=0.0558	-0.09 [-1.03 to 0.84]; P=0.839	EX:11, CON:7
**DLCOc (% predicted)**	79.48 [71.46 to 87.50]	88.59 [78.39 to 98.79]	84.94 [75.89 to 94.00]	9.11 [0.36 to 17.87]; P=0.0417	5.46 [-1.62 to 12.55]; P=0.126	-3.65 [-14.57 to 7.27]; P=0.504	EX:11, CON:7
**VA (L)**	5.39 [4.72 to 6.06]	5.70 [4.96 to 6.44]	5.68 [4.98 to 6.38]	0.31 [-0.13 to 0.76]; P=0.164	0.29 [-0.07 to 0.65]; P=0.111	-0.02 [-0.59 to 0.54]; P=0.931	EX:11, CON:7
**VA (% predicted)**	90.35 [83.56 to 97.15]	95.95 [87.81 to 104.09]	94.63 [87.21 to 102.05]	5.60 [-0.68 to 11.88]; P=0.0790	4.27 [-0.79 to 9.33]; P=0.0952	-1.33 [-9.23 to 6.57]; P=0.736	EX:11, CON:7
**KCOc (mmol/(min*kPA*L))**	1.31 [1.17 to 1.46]	1.37 [1.21 to 1.52]	1.36 [1.21 to 1.51]	0.05 [-0.03 to 0.14]; P=0.219	0.04 [-0.03 to 0.11]; P=0.223	-0.01 [-0.12 to 0.10]; P=0.840	EX:11, CON:7
**KCOc (% predicted)**	90.15 [81.32 to 98.98]	93.88 [84.13 to 103.63]	94.07 [84.82 to 103.32]	3.73 [-2.21 to 9.67]; P=0.210	3.92 [-0.84 to 8.68]; P=0.103	0.19 [-7.34 to 7.72]; P=0.960	EX:11, CON:7
**Absolute VO2max (L/min)**	1867.90 [1510.56 to 2225.23]	2028.97 [1595.03 to 2462.92]	2277.24 [1897.51 to 2656.97]	161.08 [-158.12 to 480.27]; P=0.312	409.34 [188.02 to 630.66]; P=0.000693	248.27 [-135.76 to 632.30]; P=0.197	EX:11, CON:7
**Relative VO2max (mL/kg/min)**	23.36 [19.96 to 26.76]	25.00 [20.63 to 29.38]	26.48 [22.79 to 30.17]	1.64 [-1.88 to 5.17]; P=0.350	3.12 [0.67 to 5.57]; P=0.0143	1.48 [-2.75 to 5.70]; P=0.482	EX:11, CON:7
**Watt max (W)**	159.90 [123.42 to 196.38]	213.69 [164.34 to 263.05]	204.34 [163.20 to 245.48]	53.80 [8.28 to 99.32]; P=0.0218	44.44 [11.81 to 77.08]; P=0.00917	-9.35 [-63.50 to 44.80]; P=0.729	EX:11, CON:7

**Chestpress 1RM (kg)**	39.50 [30.71 to 48.29]	43.38 [33.79 to 52.98]	42.93 [33.70 to 52.16]	3.88 [-1.68 to 9.44]; P=0.165	3.43 [-1.24 to 8.09]; P=0.144	-0.45 [-7.65 to 6.74]; P=0.898	EX:11, CON:7
**Legpress 1RM (kg)**	132.08 [111.47 to 152.69]	151.47 [122.87 to 180.07]	140.61 [116.88 to 164.34]	19.39 [-6.99 to 45.77]; P=0.145	8.53 [-11.34 to 28.41]; P=0.389	-10.86 [-42.80 to 21.08]; P=0.496	EX:11, CON:7
**FAS score (/50)**	26.63 [24.37 to 28.90]	23.29 [20.03 to 26.56]	22.59 [19.90 to 25.29]	-3.34 [-6.98 to 0.30]; P=0.0711	-4.04 [-7.14 to -0.94]; P=0.0121	-0.70 [-4.87 to 3.47]; P=0.737	EX:11, CON:7
**Physical functioning (/100)**	75.92 [68.01 to 83.10]	88.22 [77.27 to 100.59]	89.45 [82.03 to 94.96]	12.30 [1.08 to 26.98]	13.54 [4.07 to 23.40]	1.24 [-13.96 to 16.91]	EX:11, CON:7
**Physical role limitations (/100)**	52.15 [33.94 to 70.32]	62.06 [29.29 to 94.44]	84.47 [65.31 to 105.58]	9.92 [-20.52 to 39.40]	32.33 [7.28 to 61.50]	22.41 [-14.25 to 63.73]	EX:11, CON:7
**Emotional role limitations (/100)**	73.56 [58.41 to 87.15]	92.62 [82.93 to 111.47]	94.76 [81.20 to 108.75]	19.07 [7.70 to 45.92]	21.20 [1.14 to 44.78]	2.13 [-23.63 to 23.81]	EX:11, CON:7
**Energy/fatigue (/100)**	56.76 [47.55 to 65.96]	57.05 [45.35 to 68.74]	70.66 [60.28 to 81.04]	0.29 [-9.74 to 10.32]; P=0.954	13.91 [5.79 to 22.02]; P=0.00136	13.62 [1.10 to 26.13]; P=0.0337	EX:11, CON:7
**Emotional well-being (/100)**	77.90 [72.30 to 83.50]	84.32 [76.32 to 92.31]	81.46 [74.68 to 88.23]	6.41 [-1.54 to 14.36]; P=0.111	3.56 [-2.96 to 10.07]; P=0.276	-2.86 [-12.53 to 6.81]; P=0.555	EX:11, CON:7
**Social functioning (/100)**	74.01 [61.14 to 84.13]	93.45 [83.36 to 111.31]	94.14 [84.13 to 101.85]	19.44 [6.03 to 45.17]	20.13 [8.52 to 34.83]	0.69 [-24.71 to 15.65]	EX:11, CON:7
**Pain (/100)**	69.18 [55.80 to 81.03]	76.26 [57.86 to 102.02]	74.22 [62.37 to 85.90]	7.08 [-10.74 to 38.21]	5.04 [-1.50 to 22.02]	-2.04 [-27.87 to 18.72]	EX:11, CON:7

**General health (/100)**	60.70 [52.23 to 69.18]	62.64 [49.94 to 75.33]	67.16 [56.59 to 77.73]	1.93 [-11.38 to 15.25]; P=0.771	6.46 [-4.57 to 17.48]; P=0.243	4.52 [-11.37 to 20.42]; P=0.570	EX:11, CON:7
**LVESV (mL)**	48.06 [41.14 to 54.99]	40.56 [31.61 to 49.50]	45.29 [37.24 to 53.33]	-7.51 [-15.37 to 0.36]; P=0.0609	-2.78 [-9.38 to 3.83]; P=0.399	4.73 [-5.21 to 14.67]; P=0.342	EX:11, CON:7
**LVEDV (mL)**	129.83 [115.85 to 143.81]	125.68 [109.75 to 141.60]	135.72 [120.70 to 150.74]	-4.16 [-14.99 to 6.68]; P=0.441	5.89 [-3.13 to 14.91]; P=0.193	10.04 [-3.86 to 23.95]; P=0.152	EX:11, CON:7
**LVSV (mL)**	81.79 [71.64 to 91.94]	84.63 [73.11 to 96.15]	90.61 [79.73 to 101.49]	2.84 [-4.91 to 10.59]; P=0.461	8.82 [2.37 to 15.27]; P=0.00889	5.98 [-3.97 to 15.93]; P=0.231	EX:11, CON:7
**EF (%)**	62.61 [59.42 to 65.81]	68.36 [64.01 to 72.70]	65.90 [62.06 to 69.75]	5.75 [1.66 to 9.83]; P=0.00705	3.29 [-0.15 to 6.74]; P=0.0606	-2.45 [-7.57 to 2.66]; P=0.338	EX:11, CON:7
**CO (L/min)**	6.01 [5.27 to 6.75]	5.72 [4.83 to 6.61]	6.00 [5.18 to 6.82]	-0.29 [-1.00 to 0.42]; P=0.414	-0.01 [-0.60 to 0.58]; P=0.976	0.28 [-0.62 to 1.18]; P=0.534	EX:11, CON:7
**CO/BSA (L/min/m2)**	3.05 [2.79 to 3.30]	2.93 [2.59 to 3.27]	2.98 [2.68 to 3.29]	-0.12 [-0.43 to 0.20]; P=0.466	-0.06 [-0.33 to 0.21]; P=0.636	0.05 [-0.35 to 0.45]; P=0.791	EX:11, CON:7
**PER (mL/s)**	518.84 [463.97 to 573.70]	547.19 [468.18 to 626.21]	470.72 [401.96 to 539.48]	28.36 [-50.94 to 107.66]; P=0.474	-48.12 [-115.58 to 19.34]; P=0.157	-76.48 [-174.27 to 21.32]; P=0.122	EX:11, CON:7
**PFR (mL/s)**	429.08 [362.94 to 495.23]	431.78 [343.01 to 520.55]	396.16 [317.31 to 475.02]	2.70 [-79.44 to 84.84]; P=0.947	-32.92 [-102.16 to 36.32]; P=0.341	-35.62 [-138.71 to 67.48]; P=0.489	EX:11, CON:7
**LVESV/BSA (mL/m2)**	24.64 [21.69 to 27.59]	20.34 [16.32 to 24.37]	22.76 [19.21 to 26.32]	-4.29 [-8.09 to -0.50]; P=0.0276	-1.88 [-5.08 to 1.33]; P=0.242	2.42 [-2.33 to 7.17]; P=0.310	EX:11, CON:7

**LVESV/H (mL/m)**	27.81 [24.42 to 31.20]	22.91 [18.27 to 27.55]	26.20 [22.10 to 30.29]	-4.90 [-9.29 to -0.51]; P=0.0297	-1.61 [-5.32 to 2.10]; P=0.384	3.29 [-2.20 to 8.78]; P=0.233	EX:11, CON:7
**LVEDV/BSA (mL/m2)**	66.21 [60.66 to 71.76]	63.52 [56.95 to 70.09]	67.40 [61.30 to 73.50]	-2.69 [-7.63 to 2.26]; P=0.277	1.19 [-2.94 to 5.31]; P=0.562	3.87 [-2.45 to 10.19]; P=0.222	EX:11, CON:7
**LVEDV/H (mL/m)**	75.02 [68.42 to 81.62]	72.01 [64.13 to 79.89]	78.17 [70.88 to 85.46]	-3.00 [-9.04 to 3.04]; P=0.320	3.15 [-1.89 to 8.20]; P=0.213	6.15 [-1.56 to 13.87]; P=0.115	EX:11, CON:7
**LVSV/BSA (mL/m2)**	41.59 [37.39 to 45.79]	42.75 [37.92 to 47.57]	44.83 [40.30 to 49.37]	1.16 [-2.21 to 4.53]; P=0.490	3.25 [0.44 to 6.06]; P=0.0249	2.09 [-2.24 to 6.41]; P=0.334	EX:11, CON:7
**LVSV/H (mL/m)**	47.23 [42.04 to 52.42]	48.62 [42.60 to 54.65]	52.18 [46.55 to 57.82]	1.39 [-2.93 to 5.72]; P=0.517	4.95 [1.35 to 8.56]; P=0.00859	3.56 [-1.98 to 9.10]; P=0.201	EX:11, CON:7
**LVMASS (g)**	109.09 [98.54 to 119.64]	103.82 [92.28 to 115.36]	108.92 [97.85 to 119.99]	-5.27 [-12.01 to 1.47]; P=0.121	-0.17 [-5.77 to 5.43]; P=0.951	5.10 [-3.58 to 13.78]; P=0.241	EX:11, CON:7
**LVMASS/BSA (g/m2)**	55.17 [51.18 to 59.16]	53.22 [48.37 to 58.08]	53.74 [49.28 to 58.20]	-1.95 [-5.82 to 1.92]; P=0.313	-1.43 [-4.67 to 1.80]; P=0.375	0.52 [-4.41 to 5.45]; P=0.832	EX:11, CON:7
**LVMASS/H (g/m)**	62.90 [56.96 to 68.84]	61.23 [54.43 to 68.04]	62.66 [56.25 to 69.06]	-1.67 [-6.38 to 3.03]; P=0.475	-0.25 [-4.17 to 3.67]; P=0.898	1.42 [-4.61 to 7.46]; P=0.635	EX:11, CON:7
**T1 global (msec)**	1026.73 [1016.57 to 1036.89]	1019.78 [1003.31 to 1036.26]	1007.85 [994.06 to 1021.64]	-6.95 [-24.49 to 10.59]; P=0.427	-18.88 [-33.59 to -4.17]; P=0.0134	-11.93 [-33.06 to 9.20]; P=0.261	EX:11, CON:7
**T2 global (msec)**	48.20 [47.54 to 48.97]	48.07 [46.83 to 49.48]	48.03 [47.14 to 49.03]	-0.14 [-1.30 to 1.38]	-0.17 [-1.24 to 0.80]	-0.03 [-1.62 to 1.57]	EX:11, CON:7

**Psychological (/100)**	76.34 [67.82 to 84.75]	90.78 [77.61 to 100.26]	88.43 [79.61 to 96.25]	14.44 [-1.20 to 24.16]	12.09 [1.10 to 24.15]	-2.35 [-14.22 to 15.48]	EX:11, CON:7
**Breathlessness & activities (/100)**	49.17 [38.78 to 59.17]	72.31 [57.08 to 87.37]	68.40 [56.93 to 82.56]	23.14 [6.26 to 34.35]	19.23 [9.77 to 33.82]	-3.91 [-18.64 to 17.04]	EX:11, CON:7
**Chest symptoms (/100)**	78.40 [70.25 to 85.97]	97.32 [93.88 to 108.39]	88.15 [73.35 to 96.29]	18.92 [13.39 to 31.85]	9.75 [-9.63 to 20.35]	-9.17 [-25.12 to -0.01]	EX:11, CON:7
**Total score (/100)**	65.58 [59.10 to 72.06]	81.15 [72.33 to 89.97]	77.36 [69.75 to 84.97]	15.57 [7.27 to 23.88]; P=0.000510	11.78 [5.02 to 18.55]; P=0.00115	-3.79 [-14.02 to 6.44]; P=0.459	EX:11, CON:7
